# Supplementary material for: Systematics and Phylogenetic Interrelationships of the Enigmatic Late Jurassic Shark Protospinax annectans Woodward, 1918 with Comments on the Shark–Ray Sister Group Relationship
Source: Diversity (Basel). Author manuscript; Available in PMC 2023 Mar 21. (PMC7614347; doi:10.3390/d15030311)
Supplement: Character List [file EMS171120-supplement-Character_List.pdf]

Supplementary Materials:

Systematics and Phylogenetic Interrelationships of the Enigmatic Late Jurassic Shark *Protospinax annectans* Woodward, 1918 with Comments on the Shark-Ray Sister Group Relationship

Patrick L. Jambura [1,2,*], Eduardo Villalobos-Segura [1], Julia Türtscher [1,2], Arnaud Begat [1,2], Manuel Andreas Staggl [1,2], Sebastian Stumpf [1], René Kindlimann [3], Stefanie Klug [4], Frederic Lacombat [5], Burkhard Pohl [5,6], John G. Maisey [7], Gavin J. P. Naylor [8] and Jürgen Kriwet [1,2]

[1] University of Vienna, Department of Palaeontology, Josef-Holaubek-Platz 2, 1090 Vienna, Austria
[2] University of Vienna, Vienna Doctoral School of Ecology and Evolution (VDSEE), Djerassiplatz 1, 1030 Vienna, Austria
[3] Haimuseum und Sammlung R. Kindlimann, 8607 Aathal-Seegräben, Switzerland
[4] Georg–August Universität Göttingen, 37077 Göttingen, Germany
[5] Interprospekt Group, 1724 Ferpicloz, Switzerland
[6] Wyoming Dinosaur Center, Thermopolis, WY, USA
[7] American Natural History Museum, Department of Vertebrate Paleontology, New York, NY, USA
[8] Florida Museum of Natural History, University of Florida, Gainesville, FL, USA
* Author to whom correspondence should be addressed.

This document contains **Supplementary Material S2:** Character list used in this study.

See also:

Supplementary Material S1: Character matrix (nexus file) used in this study.

Supplementary Material S3: TNT scripts.

Supplementary Material S4: PAUP scripts.

Supplementary Material S5: Logfile TNT parsimony analysis with "conservative" molecular backbone constraint.

Supplementary Material S6: Logfile TNT parsimony analysis with fully enforced molecular backbone constraint.

Supplementary Material S7: Logfile TNT unconstrained parsimony analysis.

Supplementary Material S8: Logfile PAUP unconstrained parsimony analysis.

Supplementary Material S9: Logfile PAUP maximum-likelihood analysis.

Supplementary Material Figure S1-S5.

Supplementary Table S1: Vertebral measurements of PBP-SOL-8007.

Character list:

Characters were assembled from previously published data matrices [1–27]. Asterisks indicate publications in which the characters were discussed previously. Modifications on characters made here are denoted with the respective character. Octothorpes (#) in front of numbers are used to abbreviate "character(s)".

Neurocranium (Skeleton)

1. **Sup: Rostral cartilages: [0] arise from the medial area of the trabecula only, [1] medial area of the trabecula + lamina orbitonasalis.** (#3 Villalobos Segura *et al.* [27]*).

2. **Sub: Rostral cartilage: [0] well-developed rostral plate with various degrees of contribution from the lamina orbitonasalis, [1] reaches the tip of the snout (carried by the growth of the pectoral fin, [2] reaches the tip of the snout (growth of lamina orbitonasalis to support the cephalic fins)**. (#4 Villalobos Segura *et al.* [27]*).

3. **Sup: Rostral process "rostrum": [0] absent, [1] present.** (#1 Landemaine *et al.* [13], #1(1) Klug [12], #1 de Carvalho [6], #1 Shirai [22]*). Shirai [22] coded this as a multistate character that included the absence and several states of presence. We split this character into two separate characters to better reflect the hierarchical levels of similarity (i.e., reductive coding; see Material and Methods).

4. **Sub: Rostral process "rostrum": [0] trough-like rostrum, [1] tripodal rostrum, [2] greatly reduced to medial bar.** (#1 Landemaine *et al.* [13], #1(1) Klug [12], #21 Goto [11], #1 de Carvalho [6], #1 Shirai [22]*) In our analysis, this character has been modified (see character 3; reductive coding) and an additional state was added ([2]) to incorporate the state observed in Orectolobiformes (#21 of Goto [11]).

5. **Sup: Rostral processes: [0] absent, [1] present.** (#7 Villalobos-Segura *et al.* [27]*, #32 Villalobos-Segura *et al.* [24], #29 Aschliman *et al.* [2]*).

6. **Sub. Rostral processes (proximal articulation): [0] articulated with nasal capsules, [1] continuous with chondrocranium, [2] articulated with ventral aspect of rostral cartilage.** (#8 Villalobos-Segura *et al.* [27]*).

7. **Rostral appendix: [0] absent, [1] present.** (#9 Villalobos-Segura *et al.* [25], #3 Claeson *et al.* [4], #25 Aschliman *et al.* [2], #25 McEachran & Aschliman [14], #21 McEachran *et al.* [17]*). Villalobos-Segura *et al.* [25] considered this character to be only present in Batomorphii. However, a rostral appendix can also be found in some hexanchiform and squaliform sharks ("rostral appendage" sensu Shirai [21]. We amended the coding of this character accordingly.

8. **Ethmoidal region of neurocranium: [0] not down-curved, [1] down-curved.** (#3 Landemaine *et al.* [13], #3(3) Klug [12], #9a Goto [12], #4 de Carvalho [6]*, #2 Shirai [23]*).

9. **Caudal internasal keel: [0] absent, [1] present.** (#10 Villalobos-Segura *et al.* [25]*).

10. **Rostral passage of superficial ophthalmic nerve: [0] covered, [1] open.** (#11 Villalobos-Segura *et al.* [25]*).

11. **Superficial ophthalmic nerve: [0] exits neurocranium through prootic foramen, [1] separate foramen for superficial ophthalmic nerve.** (#6 Landemaine *et al.* [13], #6 Klug [12], #7 de Carvalho [6], #9 Shirai [22]*, #5 Shirai [21]*).

12. **Precerebral fontanelle: [0] present, [1] absent.** (#142 Villalobos-Segura *et al.* [25], #23 Landemaine *et al.* [13], #23(17) Klug [12], #4 de Carvalho & Maisey [8], #13 Shirai [21]*).

13. **Precerebral fossa: [0] absent, [1] present.** (#2 & 43 Landemaine *et al.* [13], #2(2) & 43(31) Klug [12], #3 de Carvalho [6], #5 de Carvalho & Maisey [8]* , #3 Shirai [22], #14 Shirai [21]*). Shirai [21] coded this as a multistate character that included the absence and several states of presence. We split this character into two separate characters to better reflect the hierarchical levels of similarity (reductive coding; see Material & Methods). This character was present twice in Klug [12] and Landemaine *et al.* [13].

14. **Sub: precerebral fossa: [0] circular or ovoid concavity, [1] extending anteriorly and roofed to form a tube.** (#2 & 43 Landemaine *et al.* [13], #2(2) & 43(31) Klug [12], #3 Shirai [22]*).

15. **Nasal capsules: [0] laterally expanded, [1] ventro-laterally expanded, [2] anteriorly expanded, [3] trumpet-like.** (#34 Villalobos-Segura *et al.* [25]*, #34 Villalobos-Segura *et al.* [24], #44 Landemaine *et al.* [13], #10 Claeson *et al.*, #31 Aschliman *et al.* [2], #27 McEachran & Aschliman [14], #44(32) Klug [13], #23 McEachran *et al.* [17], #4 Shirai [22], #37 Nishida [18]*).

16. **Nasal capsule margin [0] straight, [1] horn like process.** (#35 Villalobos-Segura *et al.* [25], #83 Villalobos-Segura *et al.* [24], #9 Claeson *et al.* [5], #5 Brito & Dutheil [3]*).

17. **Anterior preorbital foramen: [0] located dorsally, [1] located anteriorly.** (#12 Villalobos-Segura *et al.* [25], #37 Villalobos-Segura *et al.* [24], #35 Aschliman *et al.* [2], #27 McEachran *et al.* [17], #85 Nishida [18]*)

18. **Preorbital process (nasal capsule): [0] absent, [1] present.** (modified from #13 Villalobos-Segura *et al.* [25], #33 Aschliman *et al.* [2], #16 de Carvalho & Maisey [8]*, #25 McEachran *et al.* [17]*, #33 & 34 & 35 Shirai [21], #17 Nishida [18]*). The presence of preorbital processes has been regarded as a synapomorphy for Euselachii [25]. Revision of this character revealed that the preorbital process can originate either from the nasal capsule wall or the supraorbital shelf. This is also reflected in de Carvalho & Maisey [8] and in Shirai [21], who treated this character as multistate character or different characters. We regard these states as two different characters here (#18 and #29).

19. **Ectethmoid process: [0] absent, [1] present.** (#7 Landemaine *et al.* [13], #7(7) Klug [12], #10 Shirai [22], #8 de Carvalho [6]*).

20. **Sup: Antorbital cartilage: [0] absent, [1] present.** (#23 Villalobos-Segura *et al.* [25], #8 Villalobos-Segura *et al.* [24], #5 Claeson *et al.* [5], #8 Aschliman *et al.* [2], #2 Brito & Dutheil [3], #3 Nishida [18]*).

21. **Sub: Antorbital cartilage (shape): [0] triangular shaped with regular outline, [1] variously shaped and with an irregular outline.** (#24 Villalobos-Segura *et al.* [25]*, #9 Villalobos-Segura *et al.* [24]).

22. **Sub: Antorbital cartilages (with regular outline): [0] well-developed, [1] reduced.** (#25 Villalobos-Segura *et al.* [25]*, #9 Villalobos-Segura *et al.* [24]).

23. **Sub: Anterior process of antorbital cartilage: [0] absent, [1] present.** (#26 Villalobos-Segura *et al.* [25]*, #9 Villalobos-Segura *et al.* [24]).

24. **Position of the articulation of the antorbital cartilage on nasal capsule: [0] lateral, [1] antero-lateral, [2] postero-lateral articulation.** (#110 Villalobos-Segura *et al.* [25]*, #2 de Carvalho [7]).

25. **Subnasal fenestra: [0] absent, [1] present.** (#5 Klug [12], #6 de Carvalho [6], #6 de Carvalho & Maisey [8]*, #7 Shirai [22], #15 Shirai [21]*). Compagno [26] regarded this as a synapomorphy for squaliform sharks, however, in Shirai [21] argued that it was only present in hexanchid sharks and some Squaliformes. Holmgrin [27] described the presence of this canal in *Pristiophorus*, however, Shirai [21] didn't find any evidence for the presence of this structure in this group and coded it absent, an argumentation, subsequent studies [6,8] and this study have followed. De Carvalho [6] changed the original coding of Shirai [21] for certain squaliforms. We coded this character following de Carvalho [6] and Shirai [22].

26. **Sup: Epiphyseal foramen: [0] absent, [1] present.** (#10 Goto [11]*). This structure is also referred to as posterior fontanelle in Nishida [18]. Goto [11] coded this as a multistate character that included the absence and two states of presence. We split this character into two separate characters to better reflect the hierarchical levels of similarity (reductive coding; Material & Methods).

27. **Sub: Epiphyseal foramen: [0] separated from the prefrontal fontanelle, [1] fused with the prefrontal fontanelle.** (#10 Goto [11]*).

28. **Supraorbital crest: [0] present, [1] absent.** (#20 Villalobos-Segura *et al.* [25]*, #34 Achliman *et al.* [2], #26 McEachran *et al.* [19]*, #32 Nishida [18]*).

29. **Preorbital process (supraorbital shelf): [0] absent, [1] present.** (modified from #13 Villalobos-Segura *et al.* [25], #33 Aschliman *et al.* [2], #16 de Carvalho & Maisey [8]*, #25 McEachran *et al.* [17]*, #33 & 34 & 35 Shirai [21], #17 Nishida [18]*). The presence of preorbital processes has been regarded as a synapomorphy for Euselachii [25]. Revision of this character revealed that the preorbital process can originate either from the nasal capsule wall or the supraorbital shelf. This is also reflected in de Carvalho & Maisey [8] and in Shirai [21], who treated this character as multistate character or different characters. We regard these states as two different characters here (#18 and #29).

30. **Suborbital shelf: [0] absent, [1] present.** (#40 Villalobos-Segura *et al.* [25], #3 de Carvalho & Maisey [8]*).

31. **Basal angle of the neurocranium: [0] absent, [1] present.** (#35 Landemaine *et al.* [13], #35(24) Klug [13], #4a Goto [12], #22 de Carvalho & Maisey [8]*, #47 Shirai [21]*).

32. **Basitrabecular process: [0] absent, [1] present.** (#41 Villalobos-Segura *et al.* [25], #3a Goto [12], #21 de Carvalho & Maisey [8], #44 Shirai [21]*).

33. **Sup: Postorbital process: [0] present, [1] absent.** (modified from #27 Villalobos-Segura *et al.* [25]*, #12 Claeson *et al.* [5], #32 McEachran & Aschliman [16], #7 Brito & Dutheil [3], #35 Nishida [20]*).

34. **Sub: Postorbital process: [0] reduced, [1] triangular, [2] broad and shelf-like, [3] elongated and filamentous, [4] massive and anterolaterally projecting.** (modified from #28 Villalobos-Segura *et al.* [25]*, #36 Aschliman *et al.* [2]*, McEachran *et al.* [19], #35 & #65 Nishida [20]*). Originally this character was coded as (0) narrow and (broad and shelf like). To better capture the diversity of shapes observed in postorbital processes we split the original state 0 in: (0) reduced (as observed in many batoids), (1) triangular (as observed in most Sualomorphii, e.g., *Squalus acanthias*), (3) elongated and filamentous (as observed in Carcharhinidae and Sphyrnidae), (4) massive and anterolaterally projecting (as observed in Squatinidae).

35. **Sub: Postorbital process: [0] fused with triangular process, [1] separated from triangular process.** (#29 Villalobos-Segura *et al.* [25]*, #37 Aschliman *et al.* [2], #29 McEachran *et al.* [19]*).

36. **Sub: Postorbital process: [0] projects laterally, [1] projects ventro-laterally.** (#30 Villalobos-Segura *et al.* [25]*, #38 Aschliman *et al.* [2]*, #30 McEachran *et al.* [19]*).

37. **Hyomandibular fossa: [0] posterior part of the otic region, [1] anteriorly situated in the otic region. (**#13 de Carvalho [6], #7a Goto [12], #17 Shirai [24], #1 Shirai [23]*).

38. **Hyomandibular fossa: [0] not composed of two horizontally divided cavities, [1] fossa with cavities as such.** (#13 Landemaine *et al.* [13], #13(13) Klug [13], #18 Shirai [24], #14 de Carvalho [6]*, #29 de Carvalho & Maisey [9], #54 Shirai [23]).

39. **Concavity ventral to hyomandibular fossa: [0] absent, [1] present.** (#14 Landemaine *et al.* [13], 14(14) Klug [13], #15 de Carvalho [6]*, #26 de Carvalho & Maisey [9], #19 Shirai [24], #51 Shirai [23]*).

40. **Supraotic shelf broad: [0] absent, [1] present.** (#161 Frey *et al.* [10]*)**.**

41. **Dorsal otic ridge: [0] absent, [1] present.** (#162 Frey *et al.* [10]*).

42. **Postotic process: [0] absent, [1] present.** (#141 Frey *et al.* [10]*).

43. **Sub: Jugal arch: [0] absent, [1] present.** (#22 Villalobos-Segura *et al.* [25]*, #39 Aschliman *et al.* [2], #31 McEachran *et al.* [19]*).

44. **Stapedial arch: [0] absent, [1] present.** (#22 Shimada [20]*). Originally, Shimada [20] coded the stapedial foramina. We instead coded the process surrounding the foramina as it is easier to be observed in fossil taxa.

45. **Metotic (otic-occipital) fissure: [0] absent, [1] present.** (#169 Frey *et al.* [10*])**.**

Splanchnocranium

46. **Antimeres of upper and lower jaws [0] separated, [1] fused.** (#78 Villalobos-Segura *et al.* [25]*, #40 Aschliman *et al.* [2], #32 McEachran *et al.* [19]*).

47. **Craniopalatine articulation: [0] absent, [1] present.** (#47 Landemaine *et al.* [13], #47(35) Klug [13], #11 Shirai [22]*).

48. **Sub: Craniopalatine articulation: orbitostylic: [0] absent, [1] present.** (#47 Landemaine *et al.* [13], #47(35) Klug [13], #2a Goto [12], #20 de Carvalho & Maisey [8]*, #11 Shirai [22]*, #43 Shirai [23]*).

49. **Sub: Craniopalatine articulation - ethmoidal: [0] absent, [1] present.** (#47 Landemaine *et al.* [13], #47(35) Klug [13], #11 Shirai [22]*).

50. **Postorbital articulation: [0] absent, [1] present.** (#17 Villalobos-Segura *et al.* [25]*, #11(11) Klug [13], #12 de Carvalho [7], #15 de Carvalho & Maisey [8]*, #14 Shirai [24], #32 Shirai [23]*).

51. **Oblique ridge or groove along medial face of palatoquadrate: [0] absent, [1] present.** (#88 Frey *et al.* [10]*)**.**

52. **Large otic process of the palatoquadrate: [0] absent, [1] present.** (#87 Frey *et al.* [10]*)**.**

53. **Sub: Otic process forming a quadrate flange: [0] absent, [1] present.** (#19 Villalobos-Segura *et al.* [25]*).

54. **Mandibular joint: [0] orbital region, [1] otic region, [2] occiput, [3] well behind occiput.**

55. **Meckel's cartilage: [0] not expanded medially, [1] expanded medially.** (#79 Villalobos-Segura *et al.* [25]*, #41 Aschliman *et al.* [2], #33 McEachran *et al.* [19]*).

56. **Winglike process on Meckel's cartilage: [0] absent, [1] present.** (#80 Villalobos-Segura *et al.* [25]*, #42 Aschliman *et al.* [2], #34 McEachran *et al.* [19], #86 Nishida [20]).

57. **Sustentaculum: [0] absent, [1] present.** (NEW; Motta & Wilga [28]**).** In orectolobiform shark like *Ginglymostoma cirratum*, the mandible has a semicircular ridge that projects laterally from its caudal end (=sustentaculum sensu Gegenbauer (1872)).

58. **Gill skeleton position: [0] posterior to the occipital region, [1] partly beneath otico-occipital regions.** (#140 Villalobos-Segura *et al.* [25], #29 Coates *et al.* [5]*).

59. **Sup: Spiracularis: [0] undivided, [1] divided.** (#65 Villalobos-Segura *et al.* [25]*, #85 Aschliman *et al.* [2]*, #61 McEachran *et al.* [19]).

60. **Sub: Spiracularis (if divided): [0] divided, one bundle enters the dorsal oral membrane underlying the neurocranium, [1] splits into lateral and medial bundles, with the medial bundles inserting onto the posterior surface of the Meckel?s cartilage and the lateral bundle onto the dorsal edge of the hyomandibula, [2] subdivided proximally and inserts separately into the palatoquadrate and the hyomandibula.** (#66 Villalobos-Segura *et al.* [25]*).

61. **Coracohyomandibularis: [0] single origin, [1] separate origins.** (#68 Villalobos-Segura *et al.* [25]*, #88 Aschliman *et al.* [2]*, #64 McEachran *et al.* [19]).

62. **Hyoid arch: [0] reduced, non-suspensory, having no insertion of the dorsal constrictor muscle, [1] massive, holding the mandibular arch from behind, [2] composed of reduced ventral parts (ceratohyal missing) and developed hyomandibula, the latter suspending the lower jaw directly, [3] similar to State 2, but the articulation be- tween the hyomandibula and mandible is interrupted by a ligament.** (#46 Villalobos-Segura *et al.* [25] (modified); #41 Landemaine *et al.* [13], #41(30) Klug [13], #38 de Carvalho & Maisey [8], #72 & #73 Shirai [23]).

63. **Ceratohyal spatulate or bladed anteriorly: [0] absent, [1] present.** (#55 Frey *et al.* [10]*)**.**

64. **Small cartilages associated with hyomandibular-Meckelian ligament: [0] absent, [1] present.** (#75 Villalobos-Segura *et al.* [25]*, #47 Aschliman *et al.* [2], #38 McEachran *et al.* [19]*).

65. **Hyomandibula articulates with neurocranium beneath the otic shelf: [0] absent, [1] present.** (#139 Frey *et al.* [10]*)**.**

66. **Medial section of hyomandibula: [0] narrow, [1] expanded.** (#73 Villalobos-Segura *et al.* [25]*, #44 Aschliman *et al.* [2]).

67. **Hypohyals: [0] absent, [1] present.** (#56 Frey *et al.* [10]*)**.**

68. **Pseudohyal: [0] absent, [1] present.** (#47 Villalobos-Segura *et al.* [25]*, #3 Aschliman *et al.* [2]*).

69. **Anterior separate basibranchial series: [0] present, [1] absent.** (#131 Landemaine *et al.* [13], #132(72) Klug [13], #22 de Carvalho [7], #43 de Carvalho & Maisey [8], #32 Shirai [24]*, #90 Shirai [23]*).

70. **Basihyal: [0] present, [1] absent.** (#44 Villalobos-Segura *et al*. [25]*, #48 Villalobos-Segura *et al*. [26], #48 Aschliman *et al*. [2]).

71. **Basihyal: [0] as long as broad, [1] elongated, [2] basihyal very thin, considerably wider than long (more than 3 times).** (modified from #22 & 25 Landemaine *et al*. [13], #22 & 25 Klug [13], #65 de Carvalho [7], #8 de Carvalho & Maisey [8]*).

72. **Paired basibranchials: [0] absent, [1] present.** (#135 Landemaine *et al*. [13], #136(76) Klug [13], #40 de Carvalho & Maisey [8], #80 Shirai [23]*).

73. **Hypobranchials (second- last) direction: [0] midline directed, [1] not directed towards midline.** (#38 Villalobos-Segura *et al*. [25]*). The first hypobranchial is always directed laterally, but the remaining hypobranchials can be directed towards the midline.

74. **Hypobranchials-basibranchial: [0] articulated with the basibranchial, [1] fused.** (#42 Villalobos-Segura *et al*. [25]*).

75. **First hypobranchial-basihyal: [0] separated, [1] fused [2] segmented.** (#45 Villalobos-Segura *et al*. [25]*).

76. **Fourth hypobranchial: [0] well-developed, [1] reduced.** (#39 Villalobos-Segura *et al*. [25]*).

77. **Hypobranchial bar: [0] absent, [1] present.** (#132 Landemaine *et al*. [13], #133(73) Klug [13], #23 de Carvalho [7], #33 Shirai [22]*).

78. **Pharyngobranchial blade: [0] absent, [1] present.** (#134 Landemaine *et al*. [13], #135(75) Klug [13], #1a Goto [11], #67 de Carvalho [7], #35 Shirai [22]*).

79. **Sixth branchial unit: [0] absent, [1] present.** (#129 Landemaine *et al*. [13], #130(70) Klug [13], #20 de Carvalho [6]*, #30 Shirai [24]).

80. **Seventh branchial unit: [0] absent, [1] present.** (#130 Landemaine *et al*. [13], #131(71) Klug [13], #21 de Carvalho [6]*, #31 Shirai [24], #81 Shirai [23]*).

81. **Last ceratobranchial: [0] free of scapulocoracoid, [1] articulates with scapulocoracoid.** (#43 Villalobos-Segura *et al*. [25]*, #174 Landemaine *et al*. [13], #4 Aschliman *et al*. [2], #29 Shirai [24], #86 Shirai [23]*, #5 Nishida [20]*).

82. **Posteriormost elements of dorsal gill arches: [0] not completely fused, [1] completely fused (gill "pickaxe").** (#133 Landemaine *et al*. [13], #134(74) Klug [13], #24 de Carvalho [7], #34 Shirai [24]*).

83. **Branchial electric organs: [0] absent, [1] present.** (#21 Villalobos-Segura *et al*. [25]*, #86 Aschliman *et al*. [2]).

84. **Coracohyoideus: [0] present, [1] absent.** (#70 Villalobos-Segura *et al*. [25]*, #89 Aschliman *et al*. [2], #65 McEachran *et al*. [19]).

85. **Coracohyoides (if present): [0] parallel to body axis, [1] short, [2] diagonal, [3] fused.** (#71 Villalobos-Segura *et al*. [25]*, #89 Aschliman *et al*. [2], #65 McEachran *et al*. [19]).

86. **Dorsal labial cartilages: [0] present, [1] absent.** (#138 Landemaine *et al*. [13]*, #139(79) Klug [13]).

87. **Less than two dorsal labial cartilages: [0] absent, [1] present.** (#139 Landemaine *et al*. [13], #140(80) Klug [13]).

88. **Ventral labial cartilages: [0] present, [1] absent.** (#140 Landemaine *et al*. [13], #141(81) Klug [13]).

89. **Less than two ventral labial cartilages: [0] absent, [1] present.** (#141 Landemaine *et al*. [13], #142(82) Klug [13]).

Girdles and paired fins

90. **Ventral antimeres of scapulocoracoid: [0] fused, [1] separate.** (#105 Villalobos-Segura *et al*. [25], #1 de Carvalho & Maisey [8]*, #3 Shirai [21]*).

91. **Sup: Suprascapulae: [0] absent, [1] present.** (#93 Villalobos-Segura *et al*. [25]*, #30 Goto [11], #6 Aschliman *et al*. [2]). Villalobos-Segura *et al*. [25] coded this as a multistate character that included the absence and two states of presence. We split this character into two separate characters to better reflect the hierarchical levels of similarity (reductive coding; see Material & Methods).

92. **Sub: Suprascapulae: [0] fused medially, [1] unfused medially.** (#93 Villalobos-Segura *et al*. [25]*, #30 Goto [11], #6 Aschliman *et al*. [2]).

93. **Sub: Suprascapula interaction with axial skeleton (if fused medially): [0] interacts with axials skeleton (articulated or fused), [1] free from axial skeleton.** (#94 Villalobos-Segura *et al*. [25]).

94. **Sub: Suprascapula (if interacts with axial skeleton): [0] articulates with vertebral column, [1] fused medially to synarcual, [2] fused medially and laterally to synarcual.** (#95 Villalobos-Segura *et al*. [25]*).

95. **Sup: Suprascapula-scapula articulation: [0] curved, [1] crenate, [2] ball socket, [3] straight.** (#96 Villalobos-Segura *et al*. [25]*).

96. **Sub: Crenated suprascapula (variations): [0] with lateral projections, [1] thin upper and lower lobes, [2] upper lobe wider than lower, [3] of similar size and wide.** (#97 Villalobos-Segura *et al*. [25]*).

97. **Scapular process-scapula: [0] fused, [1] articulated.** (#98 Villalobos-Segura *et al*. [25]*).

98. **Scapular process: [0] short and dorsally directed, [1] long, U-curved, posteriorly directed, [2] short postero-dorsally directed.** (#99 Villalobos-Segura *et al*. [25]*, #86 Aschliman *et al*. [2]).

99. **Scapular process: [0] without fossa, [1] with fossa.** (#100 Villalobos-Segura *et al*. [25]*).

100. **Sup: Pectoral articulation: [0] facets, [1] facets/condyles, [2] condyles.** (#101 Villalobos-Segura *et al*. [25]*).

101. **Sub: Scapulocoracoid condyles arrangement: [0] not horizontal, [1] horizontal.** (#104 Villalobos-Segura *et al*. [25]*).

102. **Sub: Mesocondyle: [0] single, [1] segmented and small, [2] forming an elongated ridge.** (#103 Villalobos-Segura *et al*. [25]*).

103. **Number of facets on the scapulacoracoid (if pectoral articulation via facets on scapulacoracoid): [0] three, [1] two.** (NEW; based on da Silva & de Carvalho [29]). Based on observations of da Silva & de Carvalho [29], the scapulacoracoid can articulate with the pectoral basals via condyles or via facets. The number of facets on the scapulacoracoid has been reported to be either two (some lamniform sharks) or three (carcharhiniform sharks). Most other elasmobranchs have condyles on the scapulacoracoid instead.

104. **Tribasal pectoral fin: [0] absent, [1] present.** (#141 Villalobos-Segura *et al.* [25]*).

105. **Pectoral basals: [0] separated into three distinct cartilages, [1] propterygium and mesopterygium fused forming a single cartilage, [2] mesopterygium and metapterygium fused into a single cartilage, [3] all three basals fused into one piece.** (#108 Landemaine *et al.* [13], #109(51) Klug [13], #39 de Carvalho [7], #63 de Carvalho & Maisey [9], #60 & #61 Shirai [24], #138, #139 & #140 Shirai [23]*). Orectolobiformes were coded based on Goto [11] and da Silva & de Carvalho [29].

106. **Anterior extension of propterygium: [0] absent, [1] present.** (#107 Villalobos-Segura *et al.* [25]*, #62 Aschliman *et al.* [2]*).

107. **First segmentation of propterygium (if propterygium extends anterior): [0] not reaching nasal capsules, [1] reaching nasal capsules, [2] beyond nasal capsules.** (#108 Villalobos-Segura *et al.* [25]*).

108. **Proximal section of propterygium: [0] does not surpass the procondyle, [1] extend behind procondyle.** (#112 Villalobos-Segura *et al.* [25], #64 Aschliman *et al.* [2]*).

109. **Pectoral propterygium: [0] articulating with radials, [1] not contacting radials.** (#109 Landemaine *et al.* [13], 110(52) Klug [13], #5a Goto [12], #40 de Carvalho [7], #63 Shirai [24], #141 Shirai [23]*).

110. **Mesopterygium with distal portion projected anteriorly: [0] absent, [1] present.** (#106 Villalobos-Segura *et al.* [25]*, Villalobos-Segura *et al.* [26]).

111. **Pectoral metapterygium: [0] simple, [1] short proximal segment present.** (#111 Landemaine *et al.* [13], #66 de Carvalho & Maisey [9], #144 Shirai [23]*).

112. **Pectoral fin radials: [0] all articulate to pterygia, [1] some articulate directly with scapulocoracoid.** (#114 Villalobos-Segura *et al.* [25]*, Villalobos-Segura *et al.* [26]).

113. **Sup: Radial calcification: [0] crustal, [1] catenated.** (#91 Villalobos-Segura *et al.* [25]*).

114. **Pectoral fin with interradial connections ("cross-braces"): [0] absent, [1] present.** (#111 Villalobos-Segura *et al.* [25]*, #67 Shirai [22]*).

115. **Pelvic girdle: [0] separated, [1] fused.** (#124 Villalobos-Segura *et al.* [25]*).

116. **Pelvic basipterygium: [0] fused to first radial, [1] separated from first radial.** (#60 Villalobos-Segura *et al.* [25]*).

117. **Lateral prepelvic process: [0] absent, [1] present.** (#117 Villalobos-Segura *et al.* [25]*, #36 McEachran & Dunn [15]*).

118. **Postpelvic processes: [0] absent, [1] present.** (#118 Villalobos-Segura *et al.* [25]*, #37 Cleason *et al.* [5]).

119. **Sup: Posterior margin of puboischiadic bar: [0] straight or anteriorly directed, [1] posteriorly directed.** (#119 Villalobos-Segura *et al.* [25]*).

120. **Sub: Anterior margin of puboischiadic bar (if posterior face straight or anteriorly directed): [0] straight, [1] anteriorly arched.** (#120 Villalobos-Segura *et al.* [25]*).

121. **Medial process of puboischiadic bar: [0] absent, [1] present.** (#121 Villalobos-Segura *et al.* [25], #70 Aschliman *et al.* [2]*).

122. **Anterior pelvic basal: [0] not directed anterolaterally, anterolaterally directed.** (#121 Landemaine *et al.* [13], #122(63) Klug [13], #67 de Carvalho & Maisey [9]*, #148 Shirai [23]*).

123. **Overdevelopment of first pelvic radial: [0] absent, [1] present.** (#122 Villalobos-Segura *et al.* [25]*).

124. **First pelvic radial (if not overdeveloped): [0] band like, [1] slightly expanded distally, articulating with several segments in a parallel fashion, [2] rod-like and articulates with a single radial segment in a series.** (#123 Villalobos-Segura *et al.* [25]*).

125. **Reduced number of cartilages between pelvic basipterygium and clasper: [0] absent, [1] present.** (#125 Villalobos-Segura *et al.* [25]*).

126. **Dorsal margin claspers cartilages: [0] lacks medial flange, [1] possesses medial flange.** (#127 Villalobos-Segura *et al.* [25], #75 Aschliman *et al.* [2]*).

127. **Ventral marginal cartilage: [0] short, [1] long.** (#10a Goto [12], #43 de Carvalho [6], #69 Shirai [24]*, #6 Shirai [23]*). Torpedo was coded as having long ventral marginal cartilages based on Capape & Desoutter (1979).

128. **Ventral terminal cartilages: [0] simple, [1] ventral terminal cartilages are free distally and forms components sentinel or is fused with ventral marginal cartilages, [2] ventral terminal cartilages folded ventrally along its long axis to form a convex flange.** (#128 Villalobos-Segura *et al.* [25], #78 Aschliman *et al.* [2]*).

129. **Ventral terminal cartilages: [0] attached over length to axial cartilages, [1] free of axial.** (#129 Villalobos-Segura *et al.* [25], #79 Aschliman *et al.* [2]*).

130. **Accessory terminal (T-3) cartilage: [0] spinous, [1] not spinous, [2] modified into the external mesorhipidion.** (#122 Landemaine *et al.* [13], #123 Klug [13], #70 Shirai [24]*).

Axial skeleton

131. **Sup.: Calcified vertebral centra: [0] absent, [1] present.** (#181 Frey *et al.* [10]).

132. **Primary calcification of vertebrae: [0] reduced or absent, [1] restricted terminally, [2] developed.** (#183 Landemaine *et al.* [13], #75 Shirai [24], #151 & 152 Shirai [23]*).

133. **Secondary calcification of vertebrae: [0] absent or poorly developed, [1] with endochordal radii radiating from the notochordal sheath, [2] with developed solid medialia and diagonal calcified lamellae, [3] compact mass.** (#184 Landemaine *et al.* [13], #76 Shirai [24], #155 Shirai [23]*). Coding for Orectolobus followed examinations in Claeson and Hilger [30]].

134. Cervical vertebra: [0] unmodified, [1] synarcual, product of expansion of vertebra centra, [2] fusion of neural/basidorsal and haemal/basiventral elements. (#48 Villalobos-Segura *et al*. [25], #5 Aschliman *et al*. [2]*).

135. Lateral stays: [0] free of medial crest, [1] fused with medial crest. (#51 Villalobos-Segura *et al*. [25]*).

136. Orientation of lateral stays: [0] dorsally directed, [1] laterally directed. (#52 Villalobos-Segura *et al*. [25], #53 Villalobos-Segura *et al*. [26]).

137. Ventral occipital-synarcual articulation: [0] synarcual lip firmly fitted into notch in basicranium, [1] synarcual lip rests in foramen magnum, [2] synarcual lip reduced, with a paired connection into notch in basicranium. (#53 Villalobos-Segura *et al*. [25]*, #52 Aschliman *et al*. [2]*).

138. Position of vertebral centra in the synarcual relative to position of suprascapula in the synarcual: [0] present the entire length, [1] reaching rostral to suprascapula, [2] reaching caudal to suprascapulas. (#55 Villalobos-Segura *et al*. [25], #78 Marrama *et al*. [15]).

139. Occipital hemicentrum: [0] absent, [1] present. (#50 Villalobos-Segura *et al*. [25], #16 Landemaine *et al*. [13], #16(16) Klug [12], #17 de Carvalho [6], #29 de Carvalho & Maisey [8], #21 Shirai [22], #53 Shirai [21]*). For the applied coding, see the Discussion section of this manuscript.

140. Expanded basiventral process of cervical vertebrae: [0] absent, [1] present. (#49 Villalobos-Segura *et al*. [25]*).

141. Number of vertebrae with expanded basiventrals (if expanded basiventrals are present): [0] one, [1] two or more, [2] fused as part of the synarcual. (NEW). According to Claeson and Hilger [30], the basiventrals in rays are fused and form part of the synarcual. We, therefore, coded the fusion of the basiventrals as a separate state.

142. Supraneurals: [0] absent or poorly developed, [1] well developed. (#62 Landemaine *et al*. [13], #62(38) & 91(39) Klug [13], #69 de Carvalho & Maisey [9]*, #156 Shirai [23]*).

143. Plate-like supraneurals (if supraneurals well developed): [0] enlarged at least in front of the second dorsal fin, [1] also enlarged in the abdominal region. (#62 & 90 Landemaine *et al*. [13], #62(38) & 91(39) Klug [13], #70 de Carvalho & Maisey [9]*, #157 Shirai [23]*).

144. Hemal arch: [0] not arched at anterior precaudal tail vertebrae, [1] almost complete in the entire region of the precaudal tail. (#96 Landemaine *et al*. [14], #97(42) Klug [13], #47 de Carvalho [7], #78 Shirai [22], #159 Shirai [23]*).

145. Hemal processes at the precaudal tail: [0] shorter than that of the lower lobe of the caudal fin, [1] elongate and equal to those of the caudal fin. (#97 Landemaine *et al*. [13], #98(43) Klug [13], #48 de Carvalho [7], #79 Shirai [24], #160 Shirai [23]*).

146. Vertebral ribs: [0] absent, [1] present. (#90 of Villalobos-Segura *et al*. [25], #44 Aschliman *et al*. [2], #105 Klug [12], #49 McEachran & Aschliman [14], #44 McEachran *et al*. [17], #74 Shirai [22], #158 Shirai [21]*, #64 Nishida [18]).

147. Pleural ribs: [0] absent, [1] present. (NEW; modified from Maisey [31]). According to Maisey [31], the "ribs" of elasmobranchs and hybodontiforms are not homologous. In hybodonts, the ribs are placed on the mesial wall of the body cavity, whereas in

elasmobranchs, the ribs are inserting in the horizontal myoseptum between epaxial and hypaxial body muscles [31].

148. Arcualia dorsalis: [0] absent, [1] present. (#54 Villalobos-Segura *et al*. [25], #30 Brito *et al*. [4]*).

149. Second synarcual: [0] absent, [1] present. (#88 Villalobos-Segura *et al*. [25], #54 Aschliman *et al*. [2], #43 McEachran *et al*. [19], #66 Nishida [20]*).

150. Caudal vertebrae: [0] diplospondylus, [1] fused. (#89 Villalobos-Segura *et al*. [25], #80 Aschliman *et al*. [2]*).

151. Number of dorsal fins: [0] two, [1] one. (#145 Landemaine *et al*. [13], #149(89) Klug [13], #50 de Carvalho [7], #83 de Carvalho & Maisey [8]*, #80 Shirai [24]).

152. Dorsal fin: [0] aplesodic, [1] plesodic. (modified from #36 Stone & Shimada [23], #35 Shimada [20]).

153. Dorsal fin endoskeleton: [0] composed of a triangular or rectangular basal cartilage and radials, [1] an elongate basal with radials, [2] radials only (no basal). (modified from #123 & #126 Landemaine *et al*. [13], #124(64) & 127(67) Klug [13], #11a Goto [12], #49 de Carvalho [7], #76 de Carvalho & Maisey [9], #81 Shirai [24], #162-166 Shirai [23]*).

154. Basal plates: [0] not anchored to vertebral column, [1] in close contact with vertebral column. (#125 Landemaine *et al*. [13], #126(66) Klug [13], #75 de Carvalho & Maisey [9]*, #164 Shirai [23]*).

155. Number of basal plates (if the endoskeleton is composed of triangular or rectangular basal cartilage): [0] one, [1] at least two large plates. (NEW). Most sharks have a single basal plate (or none). In *Squatina* and *Pristiophorus*, however, two large and several smaller basal plates were reported [8,21].

156. Anal fin: [0] absent, [1] present. (#128 Landemaine *et al*. [13], #129(69) Klug [13], #66 de Carvalho [7], #77 de Carvalho & Maisey [9], #84 Shirai [24], #167 Shirai [23]*).

157. Anal fin skeleton: [0] composed of radials only, [1] composed of basal cartilage and radials. (#127 Landemaine *et al*. [13], #128(68) Klug [13], #13a Goto [12], #51 de Carvalho [7], #85 Shirai [24], #8 Shirai [23]*).

158. Caudal fin: [0] with a developed lower lobe to make a "lunate" form, [1] heterocercal, [2] reduced to the plesodic or tail folds, [3] without any tail folds or finlet. (#86 Shirai [22]*, #56 Nishida [20]).

159. Caudal lower lobe: [0] without radials, [1] with radials. (#213 Frey *et al*. [10]).

External features

160. Cephalic lobes: [0] absent, [1] present. (#61 Villalobos-Segura *et al*. [25], #10 Aschliman *et al*. [2]*, #9 McEachran *et al*. [19]*, #96 Nishida [20]*).

161. Upper eyelid: [0] present, [1] absent. (#1 of Villalobos-Segura *et al*. [25], #1 of Aschliman *et al*. [2], #1 McEachran *et al*. [19]). In rays, the cornea is fused with the skin of the skull dorsally and therefore no upper eyelid is present in this group.

162. **Nictitating eyelid: [0] absent, [1] present.** (#25 Shimada [20]). According to Compagno [32], the nictitating eyelid is a synapomorphy for Carcharhiniformes.

163. **Nostrils: [0] separated, [1] close together.** (#36 Villalobos-Segura *et al.* [25]*).

164. **Anterior nasal lobe: [0] fails to reach mouth, [1] reaches the mouth.** (#31 Villalobos-Segura *et al.* [25], #11 Aschliman *et al.* [2]*).

165. **Anterior nasal lobe: [0] fails to cover most of the medial half of the naris, [1] well-developed.** (#32 Villalobos-Segura *et al.* [25]*, #11 Aschliman *et al.* [2]).

166. **Nasal curtain fringes: [0] absent, [1] present.** (#33 Villalobos-Segura *et al.* [25]*).

167. **Infraorbital loop of suborbital and infraorbital canals: [0] absent, [1] present.** (#81 Villalobos-Segura *et al.* [25], #21 Aschliman *et al.* [2], #15 McEachran *et al.* [19]*).

168. **Subpleural loop of the hyomandibular canal: [0] broad rounded, [1] loop forms a lateral hook, [2] lateral aspects of subpleural loop are nearly parallel.** (#82 Villalobos-Segura *et al.* [25], #22 Aschliman *et al.* [2], #16 McEachran *et al.* [19]*).

169. **Sup: Abdominal canal on coracoid bar: [0] absent, [1] present.** (#83 Villalobos-Segura *et al.* [25], #24 Aschliman *et al.* [2], #18 McEachran *et al.* [19]*).

170. **Sub: Abdominal canal on coracoid bar (if present): [0] groove-cephalic lateral line forms abdominal canal on coracoid bar, [1] pores.** (#84 Villalobos-Segura *et al.* [25]*).

171. **Scapular loops of scapular canals: [0] absent, [1] present.** (#86 Villalobos-Segura *et al.* [25], #25 Aschliman *et al.* [2], #19 McEachran *et al.* [19]*).

172. **Cephalic lateral line canals on ventral surface: [0] present, [1] absent.** (#87 Villalobos-Segura *et al.* [25], #20 Aschliman *et al.* [2], #14 McEachran *et al.* [19]).

173. **Cephalic spines: [0] absent, [1] present.** (#2 Villalobos-Segura *et al.* [25]*).

174. **Placoid scales: [0] scarce or absent, [1] present.** (#133 Villalobos-Segura *et al.* [25], #15 Aschliman *et al.* [2], #11 McEachran & Dunn [17]*).

175. **Malar and alar thornes: [0] absent, [1] present.** (#134 Villalobos-Segura *et al.* [25], #17 Aschliman *et al.* [2], #22 McEachran & Dunn [17]*).

176. **Lateral rostral dermal denticles: [0] absent, [1] present.** (#136 Villalobos-Segura *et al.* [25]).

177. **Dorsal fin spines: [0] absent, [1] present.** (#130 Villalobos-Segura *et al.* [25], #12a Goto [12], #49 de Carvalho [7]*).

178. **Number of fin spines (if dorsal fin spines present). [0] two, [1] one.** (NEW).

179. **Fin spine ornamentation: [0] absent, [1] present.** (Modified from #227 & 228 Frey *et al.* [10]).

180. **Serrated tail sting: [0] absent, [1] present.** (#132 Villalobos-Segura *et al.* [25], #14 Aschliman *et al.* [2], #13 McEachran *et al.* [19]).

Dentition

181. Three-layered enameloid structure: [0] absent, [1] present. (#159 Landemaine *et al.* [13], #163(103) Klug [13]). According to Thies [33], teeth of Protospinax annectans have a three layered enameloid.

182. Dignathic heterodonty: [0] absent or weakly developed, [1] present. (#1 Pollerspöck & Straube [19], #1 Flammensbeck *et al.* [9], #1 Adnet & Cappetta [1]).

183. Dentition type: [0] clutching type, [1] tearing type, [2] cutting type, [3] cutting-clutching, [4] crushing, [5] grinding, [6] clutching-grinding, [7] crushing-grinding. (Modified from #151-157 Landemaine *et al.* [13], #155-161 Klug [13]). Classification follows Cappetta [34].

184. (Lower jaw) tooth row arrangement: [0] diagonally, [1] linearly along the jaw margin without an imbrication, [2] linearly, teeth overlapping each other and forming a continuous cutting edge. (#188 Landemaine *et al.* [13], #36 de Carvalho & Maisey [8], #96 Shirai [24], #66 & 68 Shirai [23]*).

185. (Upper jaw) tooth row arrangement: [0] diagonally, [1] linearly along the jaw margin without an imbrication, [2] linearly, teeth overlapping each other and forming a continuous cutting edge. (#189 Landemaine *et al.* [13], #37 de Carvalho & Maisey [8], #97 Shirai [24], #67, #69 & #70 Shirai [23]*).

186. Intermediate tooth row: [0] absent, [1] present. (#160 Landemaine *et al.* [13], #164(104) Klug [13]).

187. Tooth crown: [0] with more than one well-developed cusp, [1] with only one well-developed cusp. (Modified from #10 & #45 Pollerspöck & Straube [19], #7 & #25 Flammensbeck *et al.* [9]). Pollerspöck & Straube ([19]) treated the development of one/more than one cusp in teeth of the upper and lower jaw as two separate characters. Among all examined species in this study, none showed any variation between teeth of the lower and upper jaw, which is why we merged both characters here.

188. Teeth with three slim main cusps almost equal to each other, strongly recurved: [0] absent, [1] present. (#73 Frey *et al.* [10]).

189. Labial root depression: [0] absent, [1] present. (#170 Klug [12]).

190. Lower teeth labio-lingual compression: [0] strongly compressed, [1] moderately compressed, [2] not compressed. (#8 Pollerspöck & Straube [19], #3 Flammensbeck *et al.* [9]).

191. Upper teeth labio-lingual compression: [0] strongly compressed, [1] moderately compressed, [2] not compressed. (#43 Pollerspöck & Straube [19], #27 Flammensbeck *et al.* [9]).

192. Differentiated lateral uvulae on teeth: [0] absent, [1] present. (#56 Villalobos-Segura *et al.* [25], #22 Claeson *et al.* [5]).

193. (Crown) dentine: [0] orthodentine, [1] osteodentine, [2] ortho- + osteodentine. (NEW). The tooth crown of elasmobranchs has been reported to consist of either of two dentine types or both combined [35–37]. Character coding followed observations of Denton *et al.* [38], Hampe [39], Herman *et al.* [40–44], Jambura *et al.* [35–37], Moyer *et al.* [45], and unpublished observations (e.g., in *Pseudorhina*)

194. Pulp cavity: [0] absent, [1] present. (NEW). In former analyses, the presence/absence of a pulp cavity in the root was used as character (e.g., Villalobos-Segura *et al.* [25]*). However, in several species (e.g., *Chlamydoselachus*, *Pristiophorus*, *Oxynotus*, several batoids) the pulp cavity cannot be clearly allocated to either root or crown. We therefore coded the presence or absence of a pulp cavity in general, instead of allocating it to a specific topology (root or crown).

195. Root dentine: [0] osteodentine, [1] orthodentine. (#57 Villalobos-Segura *et al.* [25], #19 Aschliman *et al.* [2]). Character coding followed observations of Denton *et al.* [38], Hampe [39], Herman *et al.* [40–44], Jambura *et al.* [35–37], Moyer *et al.* [45], and unpublished observations (e.g., in *Pseudorhina*).

196. Root vascularization: [0] anaulicorhize, [1] holaulacorhize "scyliorhinid type", [2] holaulacorhize "rhinobatoid type", [3] pseudopolyaulacorhize, [4] hemiaulacorhize, [5] polyalacorhize. (Modified from #160-165 Landemaine *et al.* [13], #164-169 Klug [13]). Character coding followed Herman *et al.* [40–42], and Cappetta [34]. Cappetta [34] mentions two stages of the holaulacorhize type following Casier [46–48]: the scyliorhinid type and the rhinobatoid type. We coded these as two different stages in our analysis.

197. Crown with labio-basal coronal extensions on mesial and distal root lobes: [0] present, [1] absent. (#193 Landemaine *et al.* [14]*).

198. Upper teeth with the main cusp [0] vertical or sub-vertical (right angle), [1] with the main cusp oblique (between 0 and 45 °), [2] with the main cusp lying down (between 45° and 90°). (#47 Pollerspöck & Straube [19], #41 Flammensbeck *et al.* [9])

199. Lower teeth with distal heel [0] absent, [1] present. (#20 Pollerspöck & Straube [19], #10 Flammensbeck *et al.* [9]).

200. Lower teeth with distal heel [0] absent, [1] present. (#55 Pollerspöck & Straube [19], #32 Flammensbeck *et al.* [9]).

201. Mesial and distal arched tooth crowns: [0] absent, [1] present. (Modified from #21 & #56 Pollerspöck & Straube [19], #11 & #33 Flammensbeck *et al.* [9]*). Flammensbeck *et al.* [9] and Pollerspöck & Straube [19] treated this character as two separate characters for teeth of the lower and upper jaw. In our sample, dignathic heterodonty did not affect this character and the coded states were therefore the same for teeth of the lower and upper jaw. Consequently, we merged both characters into one character.

202. Lower teeth - root [0] with three distinct, perpendicular and developed faces, [1] with three distinct, (not perpendicular) developed faces, [2] with two distinct, developed faces. (#22 Pollerspöck & Straube [19], #13 Flammensbeck *et al.* [9]).

203. Lower teeth - root with well developed downwards pointed lobes [0] absent, [1] present. (#25 Pollerspöck & Straube [19], #4 Flammensbeck *et al.* [9]).

204. Upper teeth - root with well developed downwards pointed lobes [0] absent, [1] present. (#60 Pollerspöck & Straube [19], #28 Flammensbeck *et al.* [9]).

205. Lower teeth - prominent lingual bulge: [0] absent, [1] present. (#26 Pollerspöck & Straube [19], #14 Flammensbeck *et al.* [9], #7 Adnet & Cappetta [1]*).

206. Upper teeth - prominent lingual bulge: [0] absent, [1] present. (#61 Pollerspöck & Straube [19], #39 Flammensbeck *et al.* [9], #7 Adnet & Cappetta [1]*).

207. Sup: Lower teeth - overlapping surface: [0] absent, [1] present. (#23 Pollerspöck & Straube [19], #15 Flammensbeck *et al*. [9]).

208. Sub: Lower teeth (if overlapping surface present): [0] developed till the crown-root borderline (teeth arranged in a row, not overlapping), [1] developed on all the root` height (teeth arranged imbricative, overlapping). (#23 Pollerspöck & Straube [19], #15 Flammensbeck *et al*. [9]).

209. Upper teeth - overlapping surface: [0] absent, [1] present. (#58 Pollerspöck & Straube [19], #34 Flammensbeck *et al*. [9], Adnet & Cappetta [1]*).

210. Lower teeth - one central, prominent foramen on the labial face: [0] absent, [1] present. (#27 Pollerspöck & Straube [19], #16 Flammensbeck *et al*. [9]).

211. Teeth with one central, prominent foramen on the lingual side: [0] absent, [1] present. (Modified from #28 & #63 Pollerspöck & Straube [19], #17 & #36 Flammensbeck *et al*. [9]). Flammensbeck *et al*. [9] and Pollerspöck & Straube [19] treated this character as two separate characters for teeth of the lower and upper jaw. In our sample, dignathic heterodonty did not affect this character and the coded states were therefore the same for teeth of the lower and upper jaw. Consequently, we merged both characters into one character.

212. Teeth with one central, prominent foramen on the basal side: [0] absent, [1] present. (Modified from #29 Pollerspöck & Straube [19], #6 & #30 Flammensbeck *et al*. [9]). Flammensbeck *et al*. [9] treated this character as two separate characters for teeth of the lower and upper jaw, seemingly to avoid polystates (i.e., presence of the foramen in upper teeth, but not in teeth of the lower jaw). However, dignathic heterodonty was neither affecting this character in their, nor in our analysis. To avoid double coding of the same character, we merged both characters into one. Flammensbeck *et al*. [9] coded the presence of a single, central, prominent foramen on the lingual root face of *Pliotrema warren*. However, Herman *et al*. [40] illustrated the presence of several, smaller foramina on the basal root face. The original coding of Flammensbeck *et al*. [9] was amended here accordingly.

213. Teeth with several, prominent foramina on the labial face: [0] absent, [1] present. (Modified from 30 & #65 Pollerspöck & Straube [19], #19 & #37 Flammensbeck *et al*. [9]). Flammensbeck *et al*. [9] and Pollerspöck & Straube [19] treated this character as two separate characters for teeth of the lower and upper jaw. In our sample, dignathic heterodonty did not affect this character and the coded states were therefore the same for teeth of the lower and upper jaw. Consequently, we merged both characters into one character.

214. Lower teeth - several lateral prominent foramina on the lingual face: [0] absent, [1] present. (#31 Pollerspöck & Straube [19], #20 Flammensbeck *et al*. [9]).

215. Upper teeth - several lateral prominent foramina on the lingual face: [0] absent, [1] present. (#66 Pollerspöck & Straube [19], #38 Flammensbeck *et al*. [9]).

216. Lower teeth - button hole (= 'boutonnière') between labial and lingual face: [0] absent, [1] present. (#32 Pollerspöck & Straube [19], #18 Flammensbeck *et al*. [9]).

217. Lower teeth - lingual groove at the level of the basal part of the lingual face: [0] absent, [1] present. (#34 Pollerspöck & Straube [19], #21 Flammensbeck *et al*. [9]).

218. Lower teeth - labial groove at the level of the basal part of the lingual face: [0] absent, [1] present. (#35 Pollerspöck & Straube [19], #22 Flammensbeck *et al*. [9]).

219. **Sup: Lower teeth - uvula [0] absent, [1] present.** (#36 Pollerspöck & Straube [19], #23 Flammensbeck *et al*. [9]). Pollerspöck & Straube [19] and Flammensbeck *et al*. [9] coded this as a multistate character that included the absence and several states of presence. We split this character into two separate characters to better reflect the hierarchical levels of similarity (i.e., reductive coding; see Material and Methods).

220. **Sub: Lower teeth - uvula shape (if uvula present): [0] boils down to a convex and madly marked crown-root boundary, [1] well and strongly developed.** (#36 Pollerspöck & Straube [19], #23 Flammensbeck *et al*. [9]). Pollerspöck & Straube [19] and Flammensbeck *et al*. [9] coded this as a multistate character that included the absence and several states of presence. We split this character into two separate characters to better reflect the hierarchical levels of similarity (i.e., reductive coding; see Material and Methods).

221. **Sup: Lower teeth - apron: [0] absent, [1] present.** (#38 Pollerspöck & Straube [19], #24 Flammensbeck *et al*. [9]). Pollerspöck & Straube [19] and Flammensbeck *et al*. [9] coded this as a multistate character that included the absence and several states of presence. We split this character into two separate characters to better reflect the hierarchical levels of similarity (i.e., reductive coding; see Material and Methods).

222. **Sub: Lower teeth - apron shape (if apron present): [0] narrow, short, thick (conical), uniform, not overhanging the basal face of the root, [1] narrow, short, thin, uniform, not overhanging the basal face of the root, [2] wide, short, thin, uniform, not overhanging the basal face of the root, [3] narrow, high extended below the half root's height, thick (conical), uniform, not overhanging the basal face of the root, [4] narrow, high extended below the half root's height, thick (conical), uniform, overhanging the basal face of the root, [5] narrow, high extended below the half root's height, thin, uniform, not overhanging the basal face of the root, [6] wide, high extended below the half root's height, thick (conical), uniform, not overhanging the basal face of the root, [7] wide, high extended below the half root's height, thin, uniform, not overhanging the basal face of the root, [8] wide, high extended below the half root?s height, thin, bilobed, not overhanging the basal face of the root.** (#38 Pollerspöck & Straube [19], #24 Flammensbeck *et al*. [9]).

223. **Tooth root with a basal groove [0] absent, [1] present.** (#33 & #67 Pollerspöck & Straube [19], #5 & #29 Flammensbeck *et al*. [9]).

224. **Upper teeth: labial face with slightly plicated or ornamented crown enameloid.** (#40 Flammensbeck *et al*. [9]).

References

1. Adnet, S.; Cappetta, H. A Palaeontological and Phylogenetical Analysis of Squaliform Sharks (Chondrichthyes: Squaliformes) Based on Dental Characters. *Lethaia* **2001**, *34*, 234–248, doi:10.1080/002411601316981188.
2. Aschliman, N.C.; Claeson, K.M.; McEachran, J.D. Phylogeny of Batoidea. In *Biology of Sharks and Their Relatives*; CRC Press, 2012; pp. 57–95 ISBN 978-0-429-10654-5.
3. Brito, P.M.; Dutheil, D.B. A Preliminary Systematic Analysis of Cretaceous Guitarfishes from Lebanon. In *Mesozoic Fishes 3 - Systematics, Paleoenvironments and Biodiversity*; Arratia, G., Tintori, A., Eds.; Verlag Dr. Friedrich Pfeil: Munich, Germany, 2004; Vol. 3, pp. 101–108.

4. Claeson, K.M.; Underwood, C.J.; Ward, D.J. †*Tingitanius tenuimandibulus*, a New Platyrhinid Batoid from the Turonian (Cretaceous) of Morocco and the Cretaceous Radiation of the Platyrhinidae. *J. Vertebr. Paleontol.* **2013**, *33*, 1019–1036, doi:10.1080/02724634.2013.767266.
5. Coates, M.I.; Gess, R.W.; Finarelli, J.A.; Criswell, K.E.; Tietjen, K. A Symmoriiform Chondrichthyan Braincase and the Origin of Chimaeroid Fishes. *Nature* **2017**, *541*, 208–211, doi:10.1038/nature20806.
6. de Carvalho, M.R. Higher-Level Elasmobranch Phylogeny, Basal Squaleans and Paraphyly. In *Interrelationships of fishes*; Stiassny, M.L.J., Parenti, L.R., Johnson, D.G., Eds.; Academic Press: London, UK; New York, NY; San Diego, CA, 1996; pp. 35–62.
7. de Carvalho, M.R. Late Cretaceous Thornback Ray from Southern Italy, with a Phylogenetic Reappraisal of the Platyrhinidae (Chondrichthyes: Batoidea). In *Mesozoic Fishes 3 - Systematics, Paleoenvironments and Biodiversity*; Arratia, G., Tintori, A., Eds.; Verlag Dr. Friedrich Pfeil: München, Germany, 2004; pp. 75–100.
8. de Carvalho, M.R.; Maisey, J.G. Phylogenetic Relationships of the Late Jurassic Shark *Protospinax* Woodward 1919 (Chondrichthyes: Elasmobranchii). In *Mesozoic Fishes - Systematics and Paleoecology*; Arratia, G., Viohl, G., Eds.; Verlag Dr. Friedrich Pfeil: Munich, Germany, 1996; pp. 9–46 ISBN 3-923871-90-2.
9. Flammensbeck, C.K.; Pollerspöck, J.; Schedel, F.D.B.; Matzke, N.J.; Straube, N. Of Teeth and Trees: A Fossil Tip-Dating Approach to Infer Divergence Times of Extinct and Extant Squaliform Sharks. *Zool. Scr.* **2018**, *47*, 539–557, doi:10.1111/zsc.12299.
10. Frey, L.; Coates, M.I.; Tietjen, K.; Rücklin, M.; Klug, C. A Symmoriiform from the Late Devonian of Morocco Demonstrates a Derived Jaw Function in Ancient Chondrichthyans. *Commun. Biol.* **2020**, *3*, 1–10, doi:10.1038/s42003-020-01394-2.
11. Goto, T. Comparative Anatomy, Phylogeny and Cladistic Classification of the Order Orectolobiformes (Chondrichthyes, Elasmobranchii). *Mem. Grad. Sch. Fish. Sci. Hokkaido Univ.* **2001**, *48*, 1–100.
12. Klug, S. Monophyly, Phylogeny and Systematic Position of the †Synechodontiformes (Chondrichthyes, Neoselachii). *Zool. Scr.* **2010**, *39*, 37–49, doi:10.1111/j.1463-6409.2009.00399.x.
13. Landemaine, O.; Thies, D.; Waschkewitz, J. The Late Jurassic Shark *Palaeocarcharias* (Elasmobranchii, Selachimorpha) – Functional Morphology of Teeth, Dermal Cephalic Lobes and Phylogenetic Position. *Palaeontogr. Abt. A* **2018**, *312*, 103–165, doi:10.1127/0375-0442/2018/0000/0085.
14. McEachran, J.D.; Aschliman, N. Phylogeny of Batoidea. In *Biology of Sharks and their Relatives*; Carrier, J.C., Musick, J.A., Heithaus, M.R., Eds.; CRC Press: Boca Raton, Florida, United States, 2004; pp. 79–113.
15. McEachran, J.D.; Dunn, K.A. Phylogenetic Analysis of Skates, a Morphologically Conservative Clade of Elasmobranchs (Chondrichthyes: Rajidae). *Copeia* **1998**, *1998*, 271–290, doi:10.2307/1447424.
16. McEachran, J.D.; Miyake, T. Phylogenetic Interrelationships of Skates: A Working Hypothesis (Chondrichthyes, Rajoidei). In *Elasmobranchs as living resources: advances in the biology, ecology, systematics, and the status of the fisheries*; Pratt, H.L., Gruber, S.H., Taniuchi, T., Eds.; NOAA Technical Report NMFS, 1990; pp. 285–304.
17. McEachran, J.D.; Dunn, K.A.; Miyake, T. Interrelationships of the Batoid Fishes (Chondrichthyes: Batoidea). In *Interrelationships of fishes*; Stiassny, M.L.J., Parenti, L.R., Johnson, G.D., Eds.; Academic Press: San Diego, London, 1996; pp. 63–84.
18. Nishida, K. Phylogeny of the Suborder Myliobatidoidei. *Mem. Fac. Fish. Sci. Hokkaido Univ.* **1990**, *37*, 1–108.
19. Pollerspöck, J.; Straube, N. Phylogenetic Placement and Description of an Extinct Genus and Species of Kitefin Shark Based on Tooth Fossils (Squaliformes: Dalatiidae). *J. Syst. Palaeontol.* **2022**, *19*, 1083–1096, doi:10.1080/14772019.2021.2012537.
20. Shimada, K. Phylogeny of Lamniform Sharks (Chondrichthyes: Elasmobranchii) and the Contribution of Dental Characters to Lamniform Systematics. *Paleontol. Res.* **2005**, *9*, 55–72.

21. Shirai, S. *Squalean Phylogeny: A New Framework of "Squaloid" Sharks and Related Taxa*; Hokkaido University Press, 1992; ISBN 978-4-8329-0276-3.
22. Shirai, S. Phylogenetic Interrelationships of Neoselachians. In *Interrelationships of Fishes*; Stiassny, M.L.J., Parenti, L.R., Johnson, D.G., Eds.; Academic Press: London, UK; New York, NY; San Diego, CA, 1996; pp. 9–34.
23. Stone, N.R.; Shimada, K. Skeletal Anatomy of the Bigeye Sand Tiger Shark, *Odontaspis noronhai* (Lamniformes: Odontaspididae), and Its Implications for Lamniform Phylogeny, Taxonomy, and Conservation Biology. *Copeia* **2019**, *107*, 632–652, doi:10.1643/CG-18-160.
24. Villalobos-Segura, E.; Underwood, C.J.; Ward, D.J.; Claeson, K.M. The First Three-Dimensional Fossils of Cretaceous Sclerorhynchid Sawfish: *Asflapristis cristadentis* gen. et sp. nov., and Implications for the Phylogenetic Relations of the Sclerorhynchoidei (Chondrichthyes). *J. Syst. Palaeontol.* **2019**, *17*, 1847–1870, doi:10.1080/14772019.2019.1578832.
25. Villalobos-Segura, E.; Marramà, G.; Carnevale, G.; Claeson, K.M.; Underwood, C.J.; Naylor, G.J.P.; Kriwet, J. The Phylogeny of Rays and Skates (Chondrichthyes: Elasmobranchii) Based on Morphological Characters Revisited. *Diversity* **2022**, *14*, 1–65, doi:10.3390/d14060456.
26. Compagno, L.J.V. Phyletic Relationships of Living Sharks and Rays. *Am. Zool.* **1977**, *17*, 303–322, doi:10.1093/icb/17.2.303.
27. Holmgren, N. Studies on the Head in Fishes. Embryological, Morphological, and Phylogenetical Researches. Part II. Comparative Anatomy of the Adult Selachian Skull, with Remarks on the Dorsal Fins in Sharks. *Acta Zool.* **1941**, *22*, 1–100, doi:10.1111/j.1463-6395.1941.tb00340.x.
28. Motta, P.J.; Wilga, C.D. Anatomy of the Feeding Apparatus of the Nurse Shark, *Ginglymostoma cirratum*. *J. Morphol.* **1999**, *241*, 33–60.
29. da Silva, J.P.C.B.; de Carvalho, M.R. Morphology and Phylogenetic Significance of the Pectoral Articular Region in Elasmobranchs (Chondrichthyes). *Zool. J. Linn. Soc.* **2015**, *175*, 525–568, doi:10.1111/zoj.12287.
30. Claeson, K.M.; Hilger, A. Morphology of the Anterior Vertebral Region in Elasmobranchs: Special Focus, Squatiniformes. *Foss. Rec.* **2011**, *14*, 129–140, doi:10.1002/mmng.201100003.
31. Maisey, J.G. The Anatomy and Interrelationships of Mesozoic Hybodont Sharks. *Am. Mus. Novit.* **1982**, 1–48.
32. Compagno, L.J.V. *Sharks of the Order Carcharhiniformes*; Princeton University Press, 1988; ISBN 978-0-691-08453-4.
33. Thies, D. Jurazeitliche Neoselachier aus Deutschland und S-England. *Cour. Forschunginstitut Senckenberg* **1983**, *58*, 1–116.
34. Cappetta, H. *Handbook of Paleoichthyology, Vol. 3E: Chondrichthyes - Mesozoic and Cenozoic Elasmobranchii: Teeth*; Verlag Dr. Friedrich Pfeil: Munich, Germany, 2012; p. 512;.
35. Jambura, P.L.; Pfaff, C.; Underwood, C.J.; Ward, D.J.; Kriwet, J. Tooth Mineralization and Histology Patterns in Extinct and Extant Snaggletooth Sharks, *Hemipristis* (Carcharhiniformes, Hemigaleidae)—Evolutionary Significance or Ecological Adaptation? *PLoS One* **2018**, *13*, e0200951, doi:10.1371/journal.pone.0200951.
36. Jambura, P.L.; Kindlimann, R.; López-Romero, F.; Marramà, G.; Pfaff, C.; Stumpf, S.; Türtscher, J.; Underwood, C.J.; Ward, D.J.; Kriwet, J. Micro-Computed Tomography Imaging Reveals the Development of a Unique Tooth Mineralization Pattern in Mackerel Sharks (Chondrichthyes; Lamniformes) in Deep Time. *Sci. Rep.* **2019**, *9*, 1–13, doi:10.1038/s41598-019-46081-3.
37. Jambura, P.L.; Türtscher, J.; Kindlimann, R.; Metscher, B.; Pfaff, C.; Stumpf, S.; Weber, G.W.; Kriwet, J. Evolutionary Trajectories of Tooth Histology Patterns in Modern Sharks (Chondrichthyes, Elasmobranchii). *J. Anat.* **2020**, *236*, 753–771, doi:10.1111/joa.13145.
38. Denton, J.S.S.; Maisey, J.G.; Grace, M.; Pradel, A.; Doosey, M.H.; Bart Jr, H.L.; Naylor, G.J.P. Cranial Morphology in *Mollisquama* sp. (Squaliformes; Dalatiidae) and Patterns of Cranial Evolution in Dalatiid Sharks. *J. Anat.* **2018**, *233*, 15–32, doi:10.1111/joa.12823.
39. Hampe, O. Revision of the Xenacanthida (Chondrichthyes: Elasmobranchii) from the Carboniferous of the British Isles. *Trans. R. Soc. Edinb. Earth Sci.* **2002**, *93*, 191–237, doi:10.18452/21762.

40. Herman, J.; Hovestadt-Euler, M.; Hovestadt, D.C. Contributions to the Study of the Comparative Morphology of Teeth and Other Relevant Ichthyodorulites in Living Supraspecific Taxa of Chondrichthyan Fishes. Part A: Selachii. No. 4: Order: Orectolobiformes - Families: Brachaeluridae, Ginglymostomatidae, Hemiscylliidae, Orectolobidae, Parascylhidae, Rhiniodontidae, Stegostomatidae. Order: Pristiophoriformes - Family: Pristiophoridae. Order: Squatiniformes - Family: Squatinidae. *Bull. L'Institut R. Sci. Nat. Belg. Biol.* **1992**, *62*, 193–254.
41. Herman, J.; Hovestadt-Euler, M.; Hovestadt, D.C.; Stehmann, M. Contributions to the Study of the Comparative Morphology of Teeth and Other Relevant Ichthyodorulites in Living Supra-Specific Taxa of Chondrichthyan Fishes. Part B: Batomorphii No. 1c: Order Rajiformes - Suborder Rajoidei - Family: Rajidae - Genera and Subgenera: *Arhynchobatis*, *Bathyraja Richardsoni*-Type, *Cruriraja, Irolita*, *Notoraja*, *Pavoraja* (*Insentiraja*), *Pavoraja* (*Pavoraja*), *Pseudoraja*, *Raja* (*Atlantoraja*), *Raja* (*Okamejei*) and *Rhinoraja*. *Bull. L'Institut R. Sci. Nat. Belg. Biol.* **1996**, *66*, 179–236.
42. Herman, J.; Hovestadt-Euler, M.; Hovestadt, D.C.; Stehmann, M. Contributions to the Study of the Comparative Morphology of Teeth and Other Relevant Ichthyodorulites in Living Supra-Specific Taxa of Chondrichthyan Fishes. Part B: Batomorphii No. 2: Order Rajiformes - Suborder: Pristoidei - Family: Pristidae - Genera: *Anoxypristis* and *Pristis* No. 3: Suborder Rajoidei - Superfamily Rhinobatoidea - Families: Rhinidae - Genera: *Rhina* and *Rhynchobatus* and Rhinobatidae - Genera: *Aptychotrema, Platyrhina, Platyrhinoidis, Rhinobatos, Trygonorrhina, Zanobatus* and *Zapteryx*. *Bull. Inst. R. Sci. Nat. Belg. Biol.* **1997**, *67*, 107–162.
43. Herman, J.S.; Hovestadt-Euler, M.; Hovestadt, D.C.; Stehmann, M. Contributions to the Study of the Comparative Morphology of Teeth and Other Relevant Ichthyodorulites in Living Supra-Specific Taxa of Chondrichthyan Fishes. Part B: Batomorphii 4a: Order Rajiformes – Suborder Myliobatoidei – Superfamily Dasyatoidea – Family Dasyatidae – Subfamily Dasyatinae - Genera: *Amphotistius*, *Dasyatis*, *Himantura*, *Pastinachus*, *Pteroplatytrygon*, *Taeniura*, *Urogymnus* and *Urolophoides* (Incl. Supraspecific Taxa of Uncertain Status and Validity), Superfamily Myliobatoidea – Family Gymnuridae - Genera: *Aetoplatea* and *Gymnura*, Superfamily Plesiobatoidea – Family Hexatrygonidae - Genus: *Hexatrygon*. *Bull. L'Institut R. Sci. Nat. Belg. Biol.* **1998**, *68*, 145–197.
44. Herman, J.; Hovestadt-Euler, M.; Hovestadt, D.C.; Stehmann, M. Contributions to the Study of the Comparative Morphology of Teeth and Other Relevant Ichthyodorulites in Living Supraspecific Taxa of Chondrichthyan Fishes Part B: Batomorphii 4: Order Torpediniformes - Family Narcinidae - Subfamily Narcininae - Genera: *Benthobatis*, *Diplobatis*, *Discopyge* and *Narcine*, Subfamily Narkinae - Genera: *Bengalichthys*, *Crassinarke*, *Heteronarce*, *Narke*, *Temera*, and *Typhlonarke*, Family Torpedinidae - Subfamily Torpedininae - Genus: *Torpedo* - Subgenus: *T.(Tetronarke)* and *T. (Torpedo)* and Subfamily Hypninae - Genus: *Hypnos*. *Bull. L'Institut R. Sci. Nat. Belg. Biol.* **2002**, *72*, 5–45.
45. Moyer, J.; Finucci, B.; Riccio, M.; Irschick, D. Dental Morphology and Microstructure of the Prickly Dogfish *Oxynotus bruniensis* (Squaliformes: Oxynotidae). *J. Anat.* **2020**, *237*, 916–932, doi:10.1111/joa.13251.
46. Casier, E. Constitution et évolution de Ia racine dentaire des Euselachii. I- Note préliminaire. *Bull. Inst. R. Sci. Nat. Belg. Biol.* **1947**, *23*, 1–15.
47. Casier, E. Constitution et évolution de Ia racine dentaire des Euselachii. II- Etude comparative des types. *Bull. Inst. R. Sci. Nat. Belg. Biol.* **1947**, *23*, 1–32.
48. Casier, E. Constitution et évolution de Ia racine dentaire des Euselachii. III - Evolution des principaux caractères morphologiques et conclusions. *Bull. Inst. R. Sci. Nat. Belg. Biol.* **1947**, *23*, 1–45.
