# Supplementary material for: Systematics and Phylogenetic Interrelationships of the Enigmatic Late Jurassic Shark Protospinax annectans Woodward, 1918 with Comments on the Shark–Ray Sister Group Relationship
Source: Diversity (Basel). Author manuscript; Available in PMC 2023 Mar 21. (PMC7614347; doi:10.3390/d15030311)
Supplement: Figs. S1-S5 [file EMS171120-supplement-Figs__S1_S5.pdf]

Supplementary Materials:

Systematics and Phylogenetic Interrelationships of the Enigmatic Late Jurassic Shark *Protospinax annectans* Woodward, 1918 with Comments on the Shark-Ray Sister Group Relationship

Patrick L. Jambura [1,2,*], Eduardo Villalobos-Segura [1], Julia Türtscher [1,2], Arnaud Begat [1,2], Manuel Andreas Staggl [1,2], Sebastian Stumpf [1], René Kindlimann [3], Stefanie Klug [4], Frederic Lacombat [5], Burkhard Pohl [5,6], John G. Maisey [7], Gavin J. P. Naylor [8] and Jürgen Kriwet [1,2]

[1] University of Vienna, Department of Palaeontology, Josef-Holaubek-Platz 2, 1090 Vienna, Austria
[2] University of Vienna, Vienna Doctoral School of Ecology and Evolution (VDSEE), Djerassiplatz 1, 1030 Vienna, Austria
[3] Haimuseum und Sammlung R. Kindlimann, 8607 Aathal-Seegräben, Switzerland
[4] Georg–August Universität Göttingen, 37077 Göttingen, Germany
[5] Interprospekt Group, 1724 Ferpicloz, Switzerland
[6] Wyoming Dinosaur Center, Thermopolis, WY, USA
[7] American Natural History Museum, Department of Vertebrate Paleontology, New York, NY, USA
[8] Florida Museum of Natural History, University of Florida, Gainesville, FL, USA
* Author to whom correspondence should be addressed.

This document contains **Supplementary Figures S1-S5**.

See also:

Supplementary Material S1: Character matrix (nexus file) used in this study.

Supplementary Material S2: Character list used in this study.

Supplementary Material S3: TNT scripts.

Supplementary Material S4: PAUP scripts.

Supplementary Material S5: Logfile TNT parsimony analysis with "conservative" molecular backbone constraint.

Supplementary Material S6: Logfile TNT parsimony analysis with fully enforced molecular backbone constraint.

Supplementary Material S7: Logfile TNT unconstrained parsimony analysis.

Supplementary Material S8: Logfile PAUP unconstrained parsimony analysis.

Supplementary Material S9: Logfile PAUP maximum-likelihood analysis.

Supplementary Table S1: Vertebral measurements of PBP-SOL-8007.

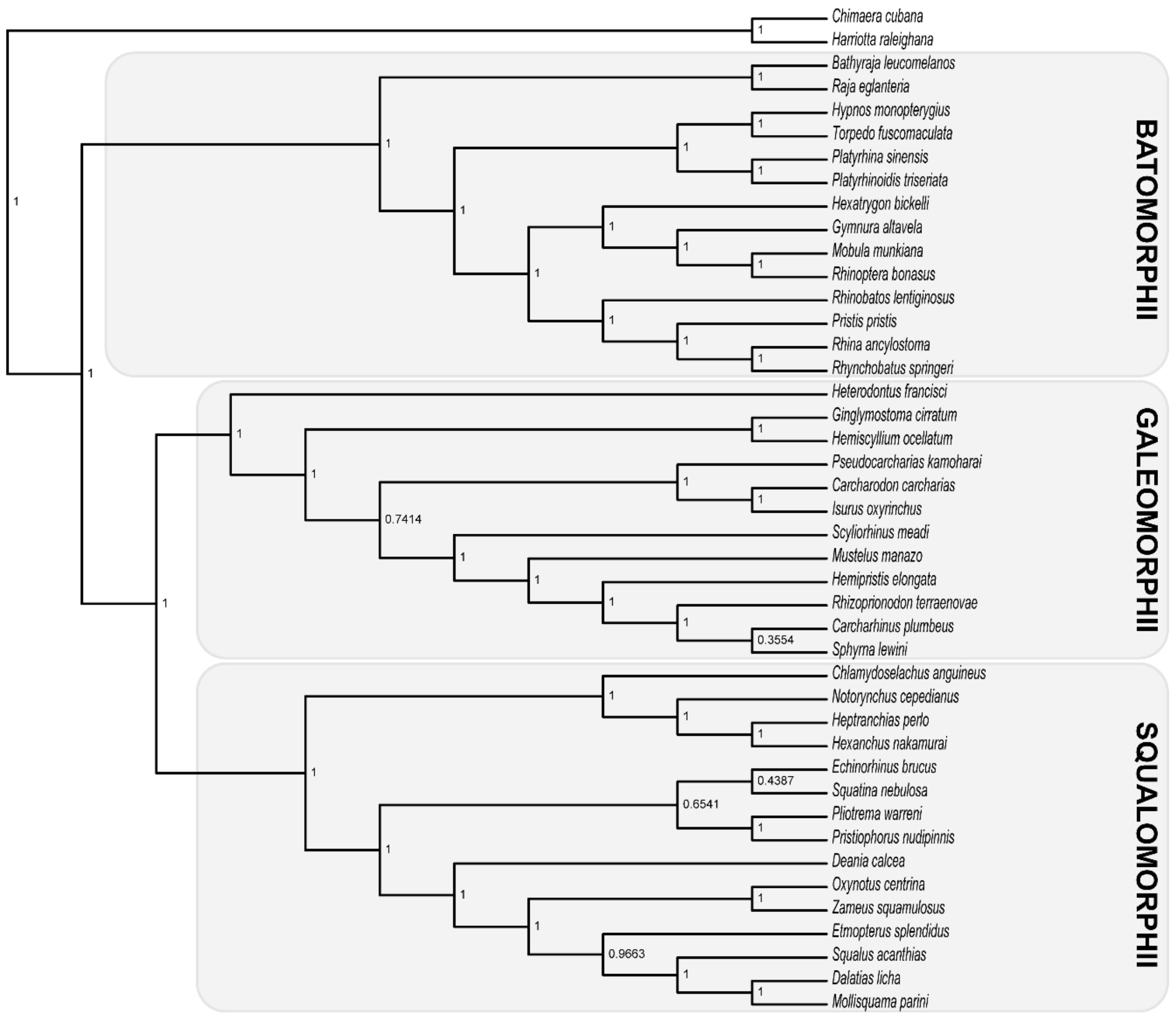

Figure S1. Molecular backbone constraint tree and posterior probability values generated from the dataset of Stein *et al.* [1].

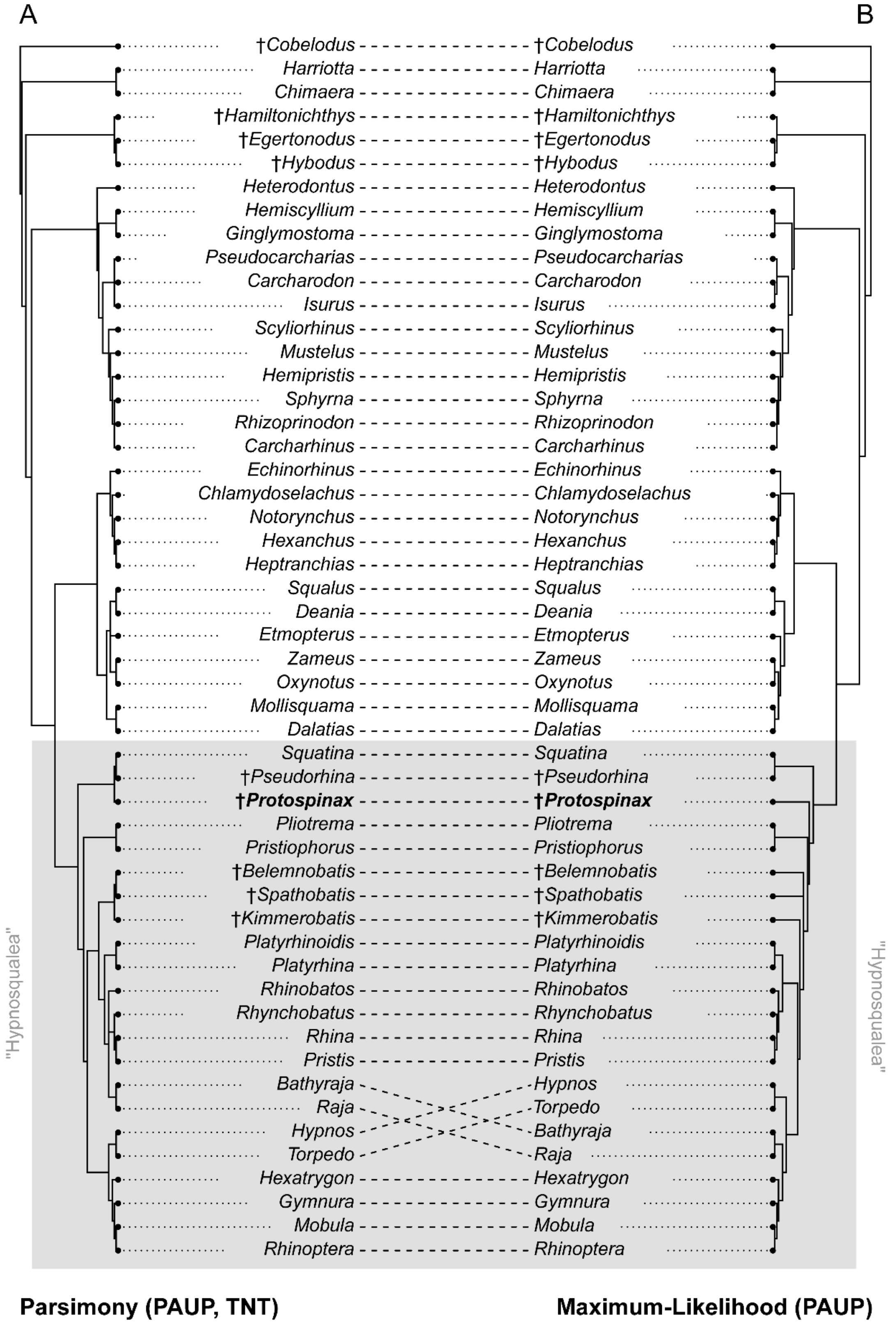

Figure S2. Phylogenetic trees obtained from two different analyses: (A) strict consensus tree from an unconstrained parsimony analysis computed in PAUP and TNT; (B) single tree generated by the (unconstrained) maximum likelihood analysis computed in PAUP.

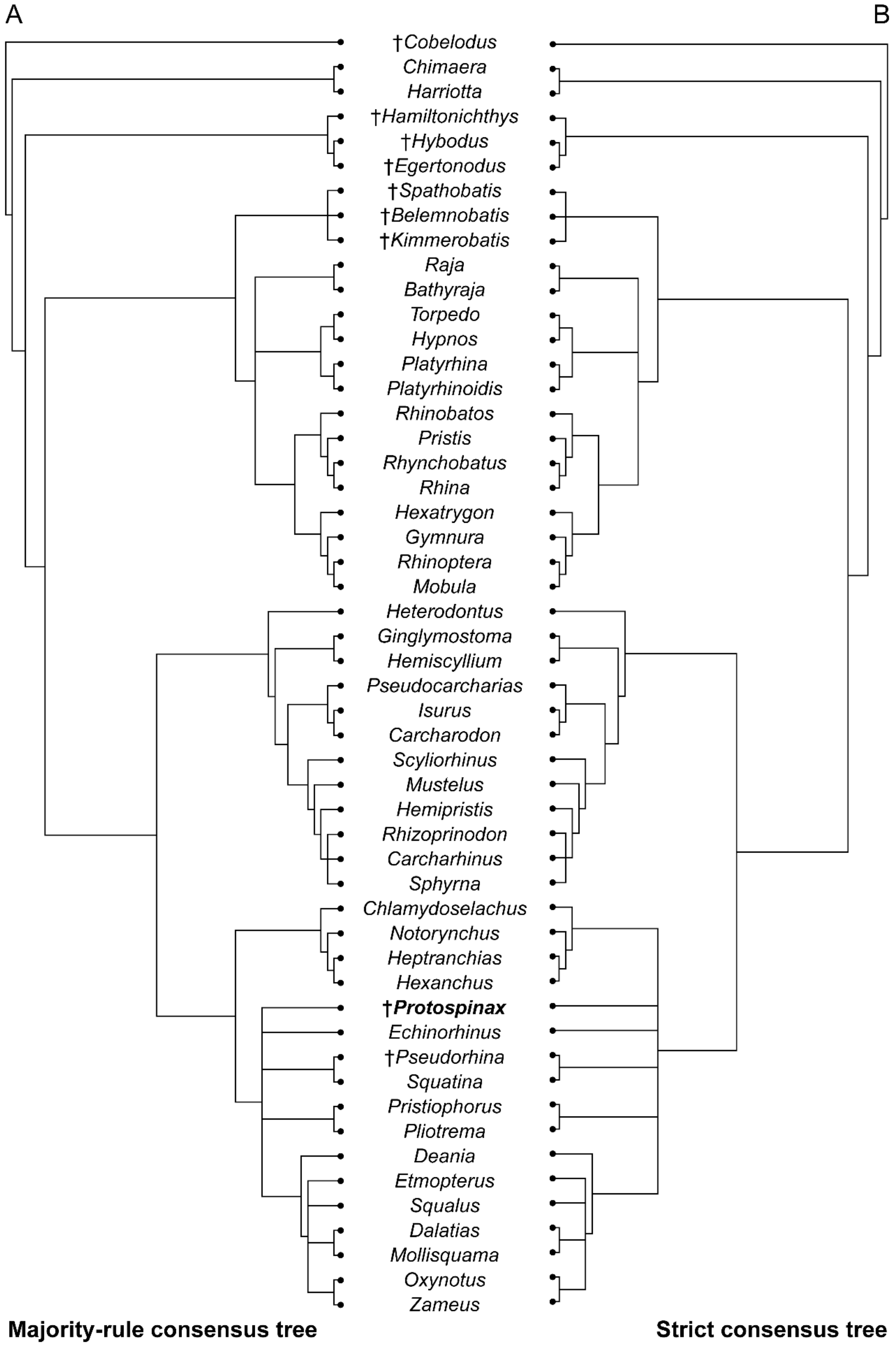

Figure S3. Majority rule consensus tree (A) and strict consensus tree (B) from a constrained parsimony analysis with fully enforced molecular backbone constraints.

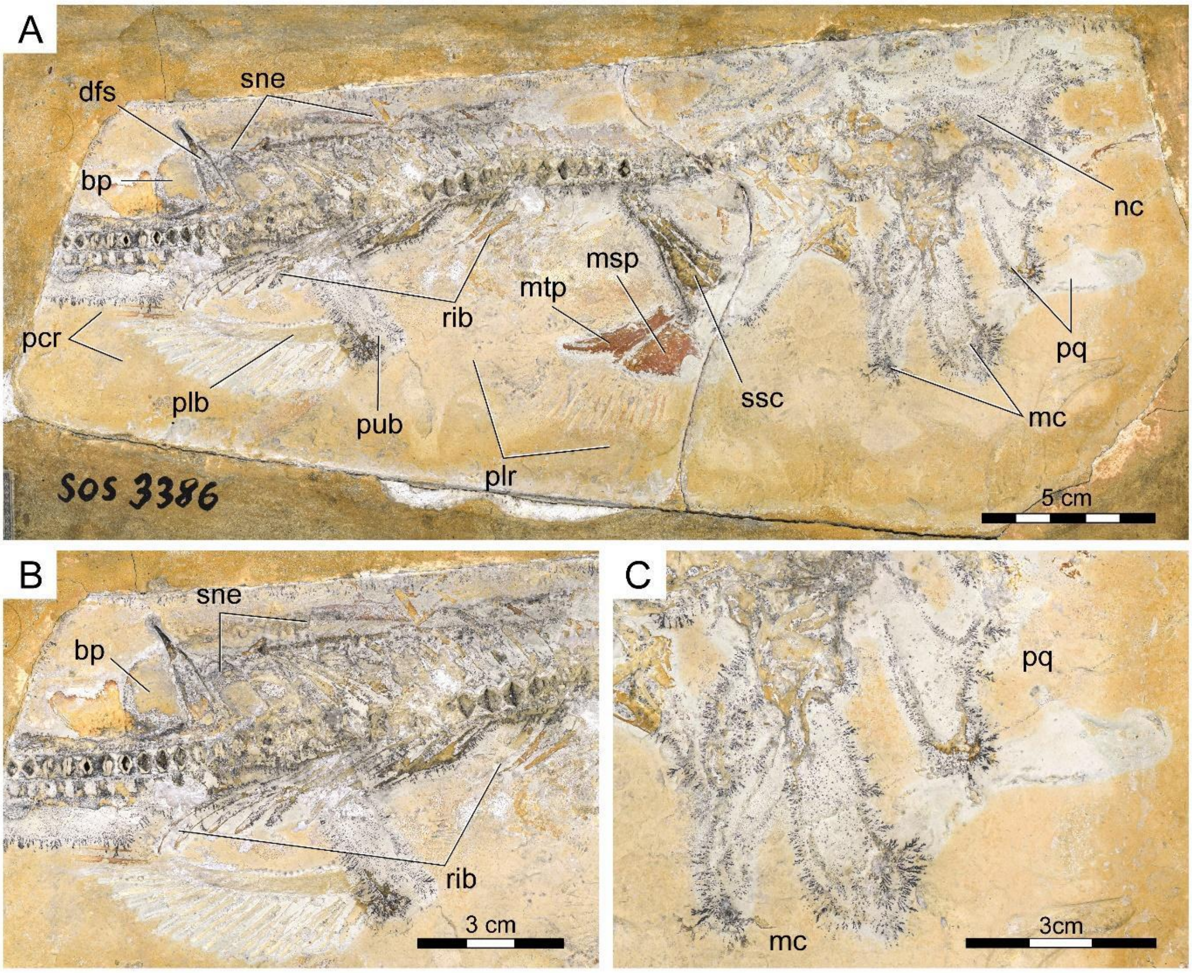

Figure S4. *Protospinax annectans* Woodward, 1918, JME-SOS 3386, from the lower Tithonian of the Solnhofen Archipelago, Bavaria, Germany. (A) whole specimen under normal light; (B) close-up view of the pelvic region; (C) close-up view of the mandibular arches. *Abbreviations*: **bp**, basal plate; **dfs**, dorsal fin spine; **mc**, Meckel's cartilage; **msp**, mesopterygium; **mtp**, metapterygium; **nc**, neurocranium; **pcr**, pectoral radial; **plb**, pelvic basipterygium; **plr**, pelvic radial; **pq**, palatoquadrate; **prp**, propterygium; **pub**, puboischiadic bar; **rib**, vertebral ribs; **scc**, scapulocoracoid; **sne**, supraneuralia.

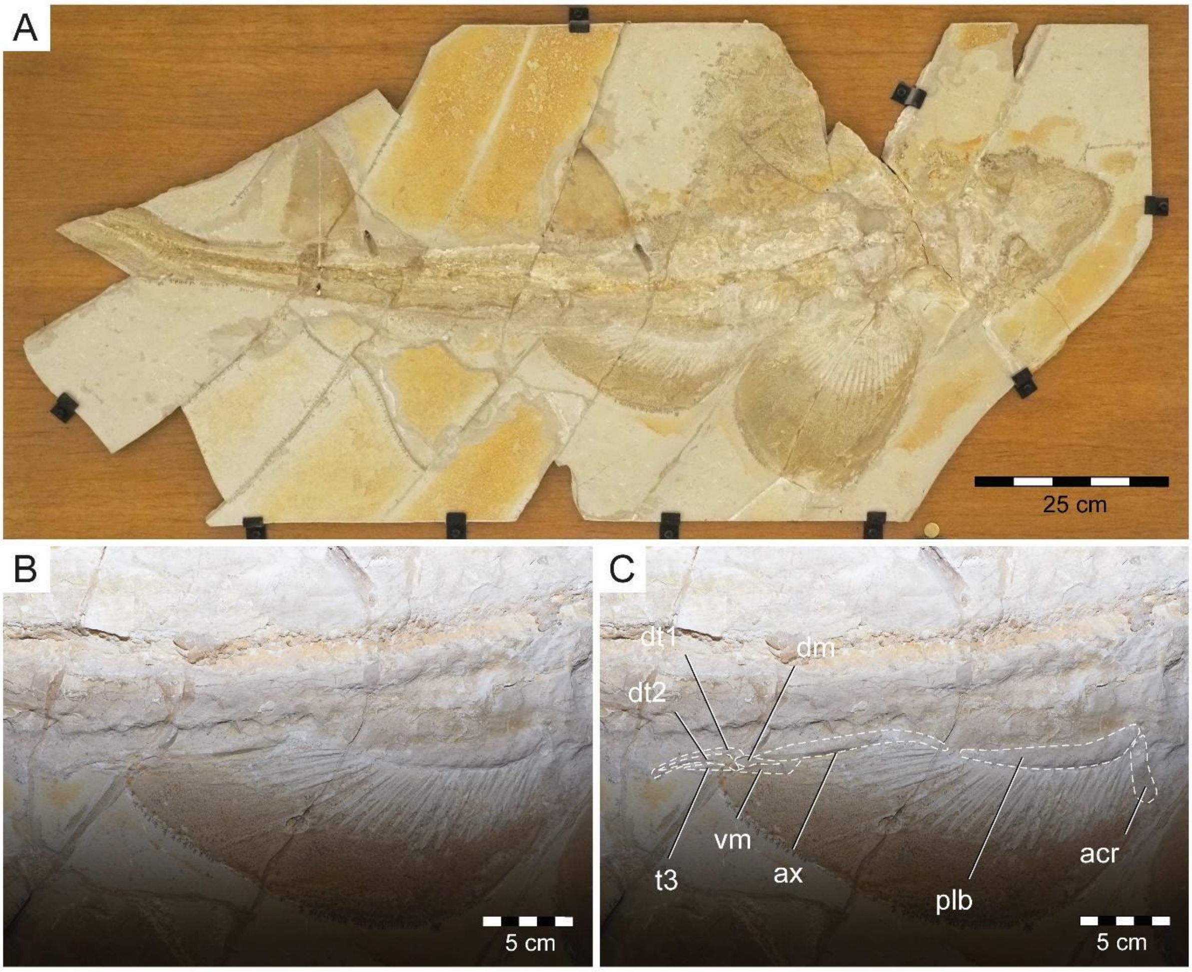

Figure S5. *Protospinax annectans* Woodward, 1918, MB 14-12-22-1, from Blumenberg, Eichstätt (Solnhofen Archipelago, lower Tithonian), Bavaria, Germany. (A) whole specimen under normal light; (B) close-up view of the pelvic region; (C) close-up view of the pelvic region with the skeletal elements of the clasper highlighted. *Abbreviation*: **acr**, anterior compound radial; **ax**, axial cartilage; **dm**, dorsal marginal cartilage; **dt1**, dorsal terminal cartilage 1; **dm2**, dorsal marginal cartilage 2; plb, pelvic basipterygium; **t3**, accessory 3 terminal cartilage; **vm**, ventral marginal cartilage.

References

1. Stein, R.W.; Mull, C.G.; Kuhn, T.S.; Aschliman, N.C.; Davidson, L.N.K.; Joy, J.B.; Smith, G.J.; Dulvy, N.K.; Mooers, A.O. Global Priorities for Conserving the Evolutionary History of Sharks, Rays and Chimaeras. *Nat. Ecol. Evol.* **2018**, *2*, 288–298, doi:10.1038/s41559-017-0448-4.
