# Supplementary material for: Systematics and Phylogenetic Interrelationships of the Enigmatic Late Jurassic Shark Protospinax annectans Woodward, 1918 with Comments on the Shark–Ray Sister Group Relationship
Source: Diversity (Basel). Author manuscript; Available in PMC 2023 Mar 21. (PMC7614347; doi:10.3390/d15030311)
Supplement: Measurements [file EMS171120-supplement-Measurements.pdf]

Table A1. Vertebral measurements of PBP-SOL-8007. Three measurements (M) were taken for the cranial-caudal length (CCL) of each vertebral centrum (V) and the average value (Av) computed.

Vertebra	CCL [mm] M1	CCL [mm] M2	CCL [mm] M3	**CCL [mm] Av**	Vertebra	CCL [mm] M1	CCL [mm] M2	CCL [mm] M3	**CCL [mm] Av**	Vertebra	CCL [mm] M1	CCL [mm] M2	CCL [mm] M3	**CCL [mm] Av**
**V1**	5.8	6.1	5.6	**5.8**	**V51**	6.8	6.4	6.8	**6.7**	**V89**	7.0	6.0	6.0	**6.3**
**V2**	6.5	5.8	5.8	**6.0**	**V52**	6.4	7.5	6.6	**6.8**	**V90**	6.5	6.0	6.3	**6.3**
**V3**	8.5	8.9	8.8	**8.7**	**V53**	7.0	6.8	6.6	**6.8**	**V91**	6.3	5.9	6.0	**6.1**
**V4**	7.8	7.6	8.0	**7.8**	**V54**	7.6	7.0	6.4	**7.0**	**V92**	5.6	5.7	5.8	**5.7**
**V5**	8.0	7.7	7.3	**7.7**	**V55**	7.4	7.1	6.7	**7.1**	**V93**	5.6	6.0	5.9	**5.8**
**V6-V20**					**V56**	6.9	6.5	6.0	**6.5**	**V94**	5.6	5.8	5.6	**5.7**
					**V57**	6.4	6.3	6.2	**6.3**	**V95**	5.7	5.5	5.5	**5.6**
					**V58**	6.3	6.6	6.9	**6.6**	**V96**	5.7	5.8	5.7	**5.7**
**V21**	7.6	7.7	7.6	**7.6**	**V59**	6.2	6.3	6.4	**6.3**	**V97**	5.5	5.2	5.0	**5.2**
**V22**	8.5	8.7	7.8	**8.3**	**V60**	6.6	6.2	6.0	**6.3**	**V98**	5.1	5.5	5.4	**5.3**
**V23**	7.5	8.2	7.7	**7.8**	**V61**	6.4	6.3	6.4	**6.4**	**V99**	5.6	5.5	5.1	**5.4**
**V24**	7.7	7.7	7.6	**7.7**	**V62**	6.3	6.0	6.1	**6.1**	**V100**	5.0	4.8	4.9	**4.9**
**V25**	7.5	8.1	8.1	**7.9**	**V63**	6.5	6.5	6.8	**6.6**	**V101**	4.9	5.1	4.9	**5.0**
**V26**	7.3	7.2	7.5	**7.3**	**V64**	5.9	6.5	6.0	**6.1**	**V102**	5.3	5.5	5.8	**5.5**
**V27**	10.3	10.2	10.7	**10.4**	**V65**	5.4	5.8	5.9	**5.7**	**V103**	5.3	5.0	4.9	**5.1**
**V28**	9.0	9.0	9.8	**9.3**	**V66**	5.8	6.1	6.1	**6.0**	**V104**	4.7	5.3	5.7	**5.2**
**V29**	8.5	8.7	8.8	**8.7**	**V67**	6.3	6.3	6.2	**6.3**	**V105**	5.6	5.6	5.7	**5.6**
**V30**	8.8	9.2	9.3	**9.1**	**V68**	6.5	6.2	6.5	**6.4**	**V106**	4.7	4.3	4.7	**4.6**
**V31**	8.3	9.6	9.0	**9.0**	**V69**	6.5	6.5	6.3	**6.4**	**V107**	4.4	4.8	4.4	**4.5**
**V32**	9.2	8.0	8.1	**8.4**	**V70**	6.6	6.5	6.7	**6.6**	**V108**	4.2	4.5	4.0	**4.2**
**V33**	7.0	7.6	7.0	**7.2**	**V71**	6.5	6.9	7.1	**6.8**	**V109**	4.7	4.1	3.5	**4.1**
**V34**	8.3	7.1	7.2	**7.5**	**V72**	6.4	6.6	6.2	**6.4**	**V110-V117**				
**V35**	7.0	6.0	7.2	**6.7**	**V73**	5.9	6.5	6.6	**6.3**					
**V36**	7.5	7.0	7.7	**7.4**	**V74**	5.7	6.2	5.8	**5.9**					
**V37**	6.2	5.8	6.4	**6.1**	**V75**	6.5	5.8	6.5	**6.3**	**V118**	3.2	3.0	2.9	**3.0**
**V38**	5.9	5.5	5.7	**5.7**	**V76**	6.6	6.4	6.4	**6.5**	**V119**	3.0	2.8	3.4	**3.1**
**V39**	6.6	6.8	6.0	**6.5**	**V77**	6.6	5.9	6.1	**6.2**	**V120**	2.6	2.8	2.7	**2.7**
**V40**	7.1	6.8	6.8	**6.9**	**V78**	6.5	6.4	6.0	**6.3**	**V121**	3.0	3.0	2.9	**3.0**
**V41**	6.7	7.5	7.0	**7.1**	**V79**	6.3	6.2	6.2	**6.2**	**V122**	2.8	3.1	2.7	**2.9**
**V42**	7.0	8.0	7.0	**7.3**	**V80**	6.4	5.9	6.0	**6.1**	**V123**	2.3	2.5	2.5	**2.4**
**V43**	6.3	7.2	6.6	**6.7**	**V81**	6.2	5.9	6.0	**6.0**	**V124**	3.0	3.0	2.8	**2.9**
**V44**	6.1	7.1	7.4	**6.9**	**V82**	6.5	6.2	6.0	**6.2**	**V125**	2.9	3.1	2.7	**2.9**
**V45**	6.6	7.0	7.6	**7.1**	**V83**	6.0	5.6	5.7	**5.8**	**V126**	2.6	2.9	2.9	**2.8**
**V46**	6.0	6.5	7.5	**6.7**	**V84**	6.3	6.5	6.1	**6.3**	**V127**	2.4	2.4	2.5	**2.4**
**V47**	5.9	6.7	6.5	**6.4**	**V85**	5.7	6.2	6.3	**6.1**	**V128**	2.1	2.1	2.2	**2.1**
**V48**	6.4	6.4	6.4	**6.4**	**V86**	6.2	5.8	5.9	**6.0**	**V129**	2.2	2.8	2.3	**2.4**
**V49**	7.5	7.0	6.6	**7.0**	**V87**	6.2	5.7	5.5	**5.8**	**V130**	2.9	2.9	2.7	**2.8**
**V50**	6.9	6.4	6.2	**6.5**	**V88**	7.0	6.2	6.4	**6.5**	**V131**	1.8	1.8	1.8	**1.8**
